# Synthesis of Bodipy-Tagged Galactoconjugates and Evaluation of Their Antibacterial Properties

**DOI:** 10.3390/molecules29102299

**Published:** 2024-05-14

**Authors:** Chiara Maria Antonietta Gangemi, Maura Monforte, Antonino Arrigo, Paola Maria Bonaccorsi, Sabrina Conoci, Antonella Iaconis, Fausto Puntoriero, Domenico Franco, Anna Barattucci

**Affiliations:** 1 Dipartimento di Scienze Chimiche, Biologiche, Farmaceutiche ed Ambientali, Università degli Studi di Messina, V.le F. Stagno D’Alcontres 31, 98166 Messina, Italy; chiaramariaantonietta.gangemi@unime.it (C.M.A.G.); maura.monforte@studenti.unime.it (M.M.); antonino.arrigo@unime.it (A.A.); paola.bonaccorsi@unime.it (P.M.B.); sabrina.conoci@unime.it (S.C.); iaconis.antonella@outlook.com (A.I.); fausto.puntoriero@unime.it (F.P.); 2 Dipartimento di Chimica “Giacomo Ciamician”, Università di Bologna, Via Francesco Selmi, 2, 40126 Bologna, Italy; 3 LAB Sense Beyond Nano—URT Department of Sciences Physics and Technologies of Matter (DSFTM) CNR, 98166 Messina, Italy

**Keywords:** BODIPY, antibacterials, galactoconjugate, luminescence

## Abstract

As a development of our research on biocompatible glycoconjugate probes and specifically multi-chromophoric systems, herein, we report the synthesis and early bactericidal tests of two luminescent glycoconjugates whose basic structure is characterized by two boron dipyrromethene difluoride (BODIPY) moieties and three galactoside rings mounted on an oligophenylene ethynylene (OPE) skeleton. BODIPY fluorophores have found widespread application in many branches of biology in the last few decades. In particular, molecular platforms showing two different BODIPY groups have unique photophysical behavior useful in fluorescence imaging. Construction of the complex architecture of the new probes is accomplished through a convergent route that exploits a series of copper-free Heck–Cassar–Sonogashira cross-couplings. The great emergency due to the proliferation of bacterial infections, in conjunction with growing antibiotic resistance, requires the production of new multifunctional drugs and efficient methods for their targeted delivery to control bacteria-associated diseases. Preliminary studies of the glycoconjugate properties as antibacterial agents against representatives of Gram-negative (*P. aeruginosa*) and Gram-positive (*S. aureus*) pathogens, which are associated with chronic infections, indicated significant bactericidal activity ascribable to their structural features.

## 1. Introduction

Luminescent glycoconjugates provide significant tools for the exploitation of biological systems. The most significant applications in the biomedical field of such molecules are (1) imaging [1], where they act as targeting probes, with the luminescent part guaranteeing great photo illumination and the sugar moiety as structural recognition element of the probe, and (2) sensing [2], where the luminescent molecule must be able to undergo changes in its lighting to act as a sensing element, with the sugar moiety improving the selectivity and solubility in aqueous media. A further development can be the design and development of fluorescent glycoconjugates for theranostic applications, in which the probe is used to image a specific cell or pathogen and, after activation, kill the target in a therapeutic approach [3].

Carbohydrates possess stereochemical and structural diversity provided by nature and synthetic chemistry [4] that constitute their imprinting in recognition phenomena and the pathological events of biological and physiological processes [5]. Mono- and oligosaccharides decorate proteins of the cellular membranes, giving access to a great variety of glycoproteins that represent a versatile molecular signal system in cells, such as in the endocytosis process. Carbohydrates’ natural widespread nature and capability of forming different kinds of chemical bonds with stereochemical differentiation expanded their use in the synthesis of luminescent probes and particularly fluorescent organic small molecule probes involved in biological investigations [6]. The rational design and feasibility of the synthesis of such probes, as well as their stable luminescence and high signal-to-noise ratio, make them an advantageous choice for in vivo imaging [7]. Additionally, carbohydrate conjugation helps to achieve low cytotoxicity and good cell permeability and targeting properties [8].

Boron Dipyrromethene difluoride (BODIPY) derivatives are usually chosen as dyes for many applications in the biological and medicinal fields [9], such as molecular imaging and luminescence-guided surgery, due to their high absorption coefficients in the visible region, good luminescence properties, high photostability, weak dependence on environmental conditions, small size, and finally their chemical versatility for *core* modification with target molecules [10]. For practical applications, the use of at least two different BODIPY derivatives on a molecular platform [11], with close proximity and significant spectral overlap, is encouraged by the high fluorescence quantum yields and large Stokes shifts of such bichromophoric species, with a high difference between the excitation and the emission energy, which allow the suppression of photon scattering and diminish tissue autofluorescence in fluorescence imaging [12].

**Figure 1:**
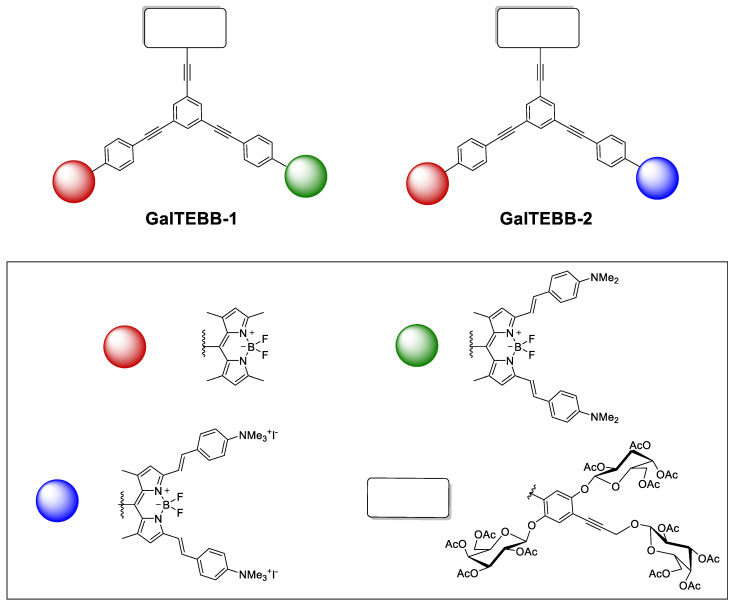
Schematic representations of **GalTEBB-1** and **GalTEBB-2**.

In continuing our research on biocompatible glycoconjugates [13] and particularly multichromophoric systems in which differently substituted BODIPY moieties, chosen to function as artificial antenna systems with extremely efficient and fast coulombic energy transfer, were linked to more than one sugar skeleton, in this paper, we describe the synthetic procedure of luminescent galactoconjugates **GalTEBB-1** and **GalTEBB-2** (Figure 1), where two BODIPY moieties and three galactoside rings are mounted onto an oligophenylenethynylene (OPE) skeletal support [14].

Apart from the bichromophoric nature of the two BODIPY derivatives, which is assessed thoroughly in this paper by through photophysical characterizations, the luminescent glycoconjugates **GalTEBB-1** and **GalTEBB-2** were conceived as derivatives targeting bacterial infections [15], with the luminescent portion constituted by the OPE and the two BODIPY residues, known in the literature as antibacterial materials [16], as well as the galactosyl moieties chosen for the capacity of several galactose-binding lectins [17,18] to modulate antibiotic activity against the *Staphylococcus* (*S.*) *aureus* and *Escherichia coli* bacterial strains [15]. New drugs and efficient strategies are required to control bacteria-associated infections due to the emergence and rapid dissemination of antibiotic resistance. To address this, various antimicrobial drugs [19] (e.g., antibacterial peptides and amphiphiles) and antibacterial materials [20] (e.g., nanoparticles [21], hydrogels, engineered surfaces [22], and surface coatings [23]) have been developed. In addition, the presence of two tetralkylammonium functionalities within the structural skeleton of one of the two BODIPY derivatives strengthened the possibility of **GalTEBB-2** acting as an antimicrobial agent against *Staphylococcus* strains, including methicillin-resistant *S. aureus* (MRSA) [24], or eradicating bacterial biofilms [25] without losing the luminescence properties of the BODIPY scaffolds.

In this paper, we report preliminary studies on the properties of **GalTEBB-1** and **GalTEBB-2** as antibacterial agents against *Pseudomonas aeruginosa* and *Staphylococcus aureus*, Gram- negative and Gram- positive representative strains, respectively [26], and common causes of nosocomial, difficult-to-eradicate infections.

## 2. Results and Discussion

### 2.1. Synthesis

With the aim to build-up the basic OPE skeleton of galactoconjugate chromophores **GalTEBB-1** and **GalTEBB-2**, two convergent routes were realized, involving the syntheses of triglycosidic moiety **6** and bichromophoric species **10**. The presence of terminal alkyne and iodoarene functionalities within all the substructures allowed the use of the copper-free Heck–Cassar–Sonogashira [26] reaction as the principal means for their connection, while paying particular attention to the molar ratios to obtain good yields and minimize the formation of side-products. We reproposed [27] the use of a multifunctionalized platform such as 1,3,5-triethynylbenzene that could guarantee a radial OPE system [28].

**Scheme 1:**
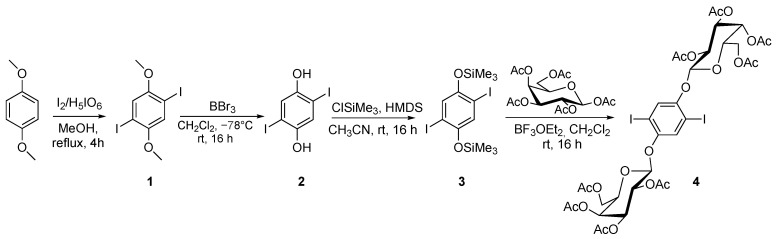
Synthethic route to digalactoside (compound **4**).

The synthetic procedure for digalactoside 4 [29] is reported in Scheme 1. Iodination of 1,4-dimethoxybenzene led to compound **1**, and subsequent treatment with BBr_3_ led to 2,5-diiodohydroquinone (compound **2**) [30]. The reaction of compound **2** with ClSiMe_3_, in the presence of HDMS, gave compound **3**, the BF_3_Et_2_O treatment of which [31], in the presence of β-D-galactopyranoside pentaacetate, afforded compound **4**, which was simply purified by crystallization from methanol. The syntheses of compound **5** [32], BDP-R 7 [33], and BDP-G 8 [34] were already reported.

**Scheme 2:**
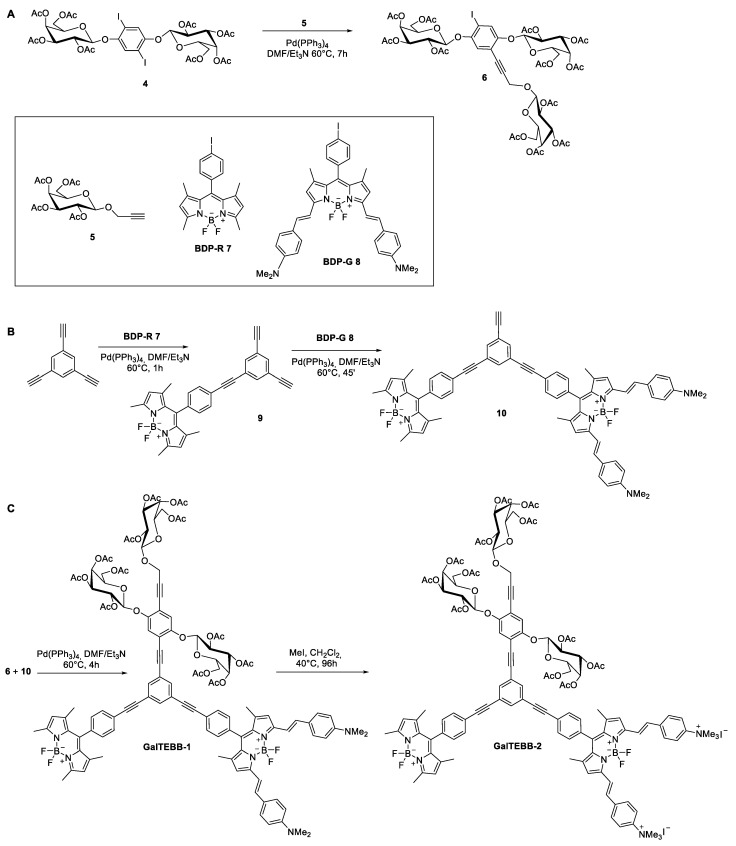
Synthetic routes to **GalTEBB-1** and **GalTEBB-2**.

Compounds **4** and **5** were cross-coupled in the presence of Pd(PPh_3_)_4_, as a catalyst, at a 2:1 stoichiometric ratio in such a way as to make the functionalization take place over only one reactive site of the ring. The reaction was complete in 7 h and, compound **6** was obtained at a 35% yield after purification (Scheme 2A). The applied conditions were the best we could experiment with to avoid side-products, even if a modest yield was achieved for compound **6**, which is attributable to the tetra-functionalization of the aromatic ring and the long but necessary reaction time. The excess of unreacted compound **4** was recovered and available to be reacted with again.

The parallel route for synthesis of bichromophoric species **10** is reported in Scheme 2B. A large excess of 1,3,5-triethynylbenzene was reacted with **BDP-R 7** in copper-free Sonogashira conditions for the obtainment of compound **9**. The use of a 1:8 stoichiometric ratio between the reactants gave the best results in terms of yield (80%). The excess of 1,3,5-triethynylbenzene was recovered as the first eluate of the column and quantitively recrystallized from hexane. The second chromophore unit **BDP-G 8** was inserted into the OPE skeleton, involving compound **9** in a further Pd(0) mediated cross-coupling, which this time was in the presence of a molar defect of **BDP-G 8**. The reaction conditions were optimized (molar ratio for compound **9** and **BDP-G 8** of 3:1) to promote the formation of compound **10** as the main product, a dark green solid, at a 76% yield. Compound **10** contained one last ethynyl arm to be easily connected with galactoconjugate (compound **6**). Therefore, once the syntheses of the precursors were completed, the next step was their assembly (Scheme 2C). An easy and straightforward copper-free cross-coupling between compounds **6** and **10** led to **GalTEBB-1** at a 47% yield after purification as a dark green solid. The conversion of **GalTEBB-1** to bis-tetraalkylammonium **GalTEBB-2** was reached using a large excess of iodomethane in DCM at reflux for three days in a sealed tube in order to keep the excess of the toxic volatile reactant and the reaction volume unchanged during the full reaction time. The reaction was quantitative, and deep blue crystals of **GalTEEB-2** were recovered by evaporation of the solvent and iodomethane in safe conditions. All the new products were fully characterized by mono- and bidimensional NMR spectroscopy and ESI-mass spectrometry to obtain their proper attributes (**ESI**, from Figures S4–S6).

### 2.2. Photophysical Characterizations

In Table 1, the photophysical characterizations of the two luminescent glycoconjugates are reported.

**Figure 2:**
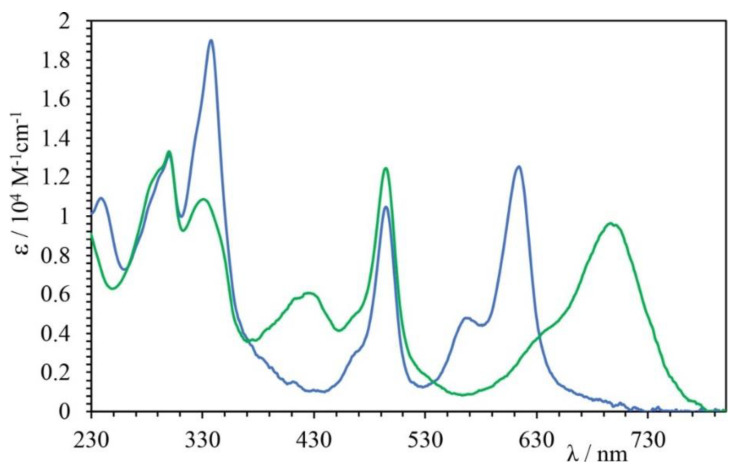
Absorption spectra of **GalTEBB-1** (green line) and **GalTEBB-2** (blue line) in CH_3_CN solution.

The absorption spectra of **GalTEBB-1** and **GalTEBB-2** in CH_3_CN are shown in Figure 2.

As can be seen, both spectra were characterized by intense absorption bands in the UV and visible regions of the electromagnetic spectrum.

In the UV region, it is possible to observe a set of bands attributable to π → π* transitions, leading to a population of S_2_ singlet states of BODIPY moieties layered over with the π → π* transitions centered on the radial OPE *core*. In the visible region, on the other hand, two different bands are distinguishable. The first one, centered at 500 nm, was due to π→ π* transitions involving the **BDP-R** moiety, whereas the contribution at lower energy (600 nm) is ascribable to the population of the S_1_ states centred on the **BPD-G** fragment in the case of **GalTEBB-2** and to π → π* transitions with a charge transfer (CT) contribution involving the **BPD-G** (700 nm) fragment for **GalTEBB-1**.

**Figure 3:**
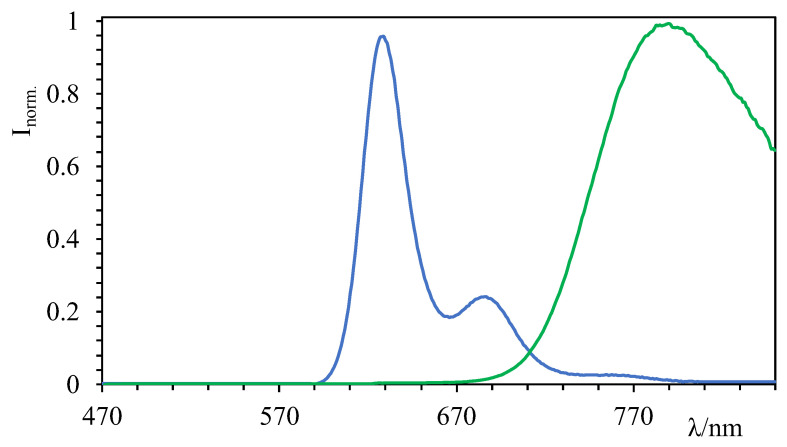
Emission spectra of **GalTEBB-1** (green line) and **GalTEBB-2** (blue line) in CH_3_CN solution.

Indeed, it is important to point out that the presence of a free electron pair on the peripheral fragment of the **BDP-G** chromophore in **GalTEBB-1** conferred a charge transfer (CT) character to lowest-lying band, which was lacking in **GalTEBB-2** due to the quaternarization of the amine group [35]. This difference between the two species was also found in the luminescence behavior. Both multicromophoric systems were emissive, as shown in Figure 3.

Independent of the excitation wavelength, **GalTEBB-1** exhibited radiative deactivation from the lower-lying excited state localized on the **BDP-G** fragment.

**Scheme 3:**
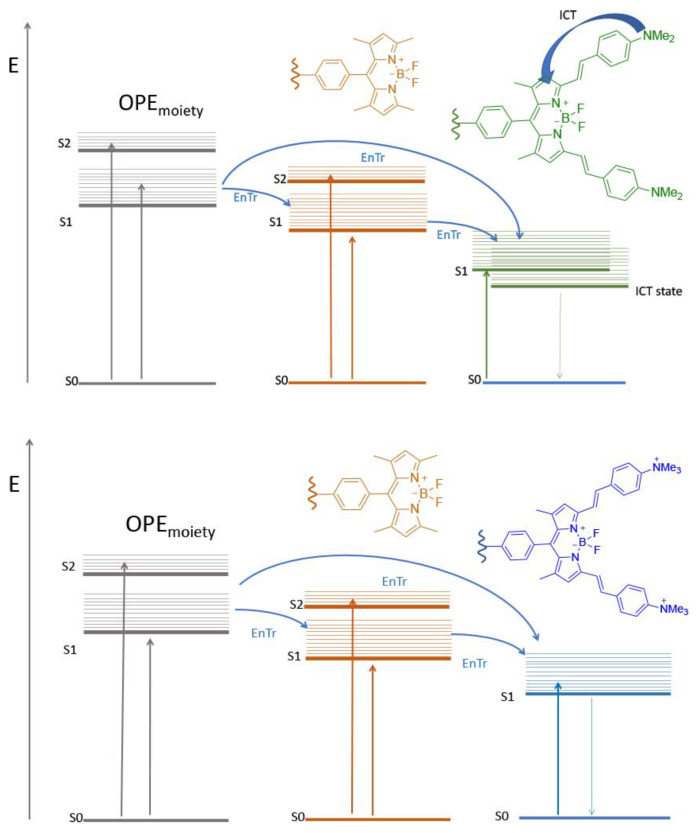
Simplified Jablonski diagram summarizing the photophysics of **GalTEBB-1** (top panel) and **GalTEBB-2** (bottom panel).

The emission spectrum was large and structureless, confirming the CT nature of the excited state. There was no evidence of residual emission from the OPE *core* or from the **BDP-R** fragment, known to be centered at 510 nm [36], suggesting that an intramolecular energy transfer process was active in this system (see Scheme 3). To confirm this behavior, excitation spectra readings at the maximum emissions (λ_max_ = 790 nm, f = 0.2, and t = 2.6 ns) were registered (see Figures S3 and S4 in the ESI), from which it is possible to state that **BDP-R** absorption also contributed efficiently to the population of the emissive state in **GalTEBB-1**.

A similar discussion can take place for **GalTEBB-2**. The emission spectrum was essentially localized at a higher energy value (l_max_ = 625 nm, f = 0.7, and t = 5.6 ns) and was much narrower and more structured with respect to the one exhibited by **GalTEBB-1**, indicating that it came from a pure p* state. Interestingly, the quantum yields for these molecules became high when amine groups were protonated or quaternarized, because in these conditions, the intramolecular charge transfer (ICT) process is not favored in an excited state [37]. Also, in this case, regardless of the excitation wavelength, the luminescence was observed exclusively from the lower-energy excited state localized on the **BDP-B** fragment. The excitation spectrum (see Figure S4 in ESI) confirmed a photoinduced energy transfer process from the **BDP-R** moiety (Scheme 3).

### 2.3. Biological Results

The bactericidal activity of **GalTEBB-1** and **GalTEBB-2** was established through biological assays against *P. aeruginosa* and *S. aureus* in the range of 15.6–250 µg/mL. Citotoxicity was also tested (Figure S5 in ESI). The lipophilic compounds were dissolved in DMSO, the antimicrobial properties of which are well known [38]. In order to avoid reporting false positive antimicrobial effects [39], we decided to also evaluate DMSO alone in the range of 0.78–12.5% (*w*/*v*), representing the proportional percentages of the organic solvent in the solutions of **GalTEBB-1** and **GalTEBB-2**, as shown in Table S1 (ESI). Thus, the bactericidal activity of **GalTEBB-1** and **GalTEBB-2** was confirmed by comparison with the data from the DMSO alone.

**Figure 4:**
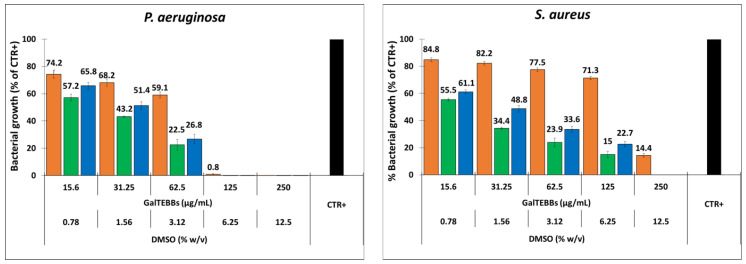
Antibacterial activity of DMSO (orange), **GalTEBB-1** (green), and **GalTEBB-2** (blue) against *P. aeruginosa* and *S. aureus.* The percentages of DMSO refer to the concentrations of the organic solvent in the **GalTEBB-1** and **GalTEBB-2** solutions (see Table S1).

The results are summarized in Figure 4.

The DMSO showed a stronger antibacterial action against *P. aeruginosa* than *S. aureus*. Regarding *P. aeruginosa*, the MIC of the DMSO was established at 6.25% (*w*/*v*), and an almost total reduction (over 99% compared with the control condition without compounds (CTR+)) of the bacterial load was observed. On the other hand, when this broth dilution was sub-cultured onto DMSO-free agar plates, the presence of viable bacteria was still observed. Therefore, the MBC was set at 3.12% (*w*/*v*). Regarding *S. aureus*, significant bacteria viability (14% compared with the control condition without compounds (CTR+)) was still observed at the highest concentration (12.5%, *w*/*v*) of DMSO. For this bacterial strain, both the MIC and MBC were found to be 25%, which are concentrations that were not used in the present work.

Based on the above, bactericidal activity was asserted on **GalTEBB-1** and **GalTEBB-2**. The data obtained after overnight (18 h) incubation (for experiments conducted after light illumination, see Figure S6 in ESI) show that both compounds exhibited significant antibacterial activity with respect to the control (bacterial culture without compounds (CTR+)), albeit to varying degrees in relation to belonging to the Gram-negative or Gram-positive group. Regarding *P. aeruginosa*, both the MIC and MBC were identified to be 125 µg/mL for both galactoconjugates. At this value, the proportional percentage of DMSO, namely 6.25% (*w*/*v*), was found for the MIC but not the MBC. These findings indicate that at these concentrations, total killing of the bacteria was obtained by the presence of the galactoconjugates. Furthermore, the optical density values at 540 nm (OD_540_) at each concentrations tested indicated a higher antibacterial effect of the galactoconjugates on bacterial growth compared with DMSO. The data were confirmed by Tukey’s multiple comparisons, for which a significant difference between the two galactoconjugates and DMSO alone was always observed, except for **GalTEBB-2** at a concentration of 15.6 µg/mL (Table S2, ESI). Concerning *S. aureus*, both the MIC and MBC were identified to be 250 µg/mL. In this case, the proportional percentage of DMSO, namely 6.25% (*w*/*v*), induced a reduction of 86% of the bacterial load, and the antibacterial activity was mainly due to **GalTEBB-1** and **GalTEBB-2**. In analogy with the results for *P. aeruginosa*, Tukey’s multiple comparisons indicated a significant difference between the two galactoconjugates and DMSO alone at the other concentrations tested (Table S3, ESI).

The MIC and MBC of **GalTEBB-1** and **GalTEBB-2** related to DMSO are summarized in Table 2.

The data indicate that both galactoconjugates had significant bactericidal activity, mostly against the Gram-negative strain. These findings could be partially due to the use of DMSO as a solvent. In fact, the action of the organic solvent on the lipopolysaccharide of the outer membrane in the Gram-negative strain could favor the uptake of the galactoconjugates. Conversely, the Gram-positive strain appeared to be less affected by the organic solvent due to its cell wall, consisting almost exclusively of peptidoglycan. Regarding the differences between the antibacterial activities of the two galactoconjugates, the data suggest that the cationic structure of **GalTEBB-2** did not boost antibacterial efficiency against the bacteria strains under study [40], as we wished.

## 3. Conclusions

In conclusion, we described a synthetic pathway, based on the copper-free Heck–Cassar–Sonogashira reaction, for the obtainment of galactoconjugates with an oligophenylenethynylene platform not lit by the presence of two BODIPY moieties. The use of the Pd-catalyzed reaction allowed the development of a convergent synthetic approach to achieve the desired compounds with satisfactory overall yields. **GalTEBB-1** and **GalTEBB-2** can be pictured as glycosyl bichromophoric probes that emit light in the red region of the visible electromagnetic spectrum, exhibiting a quite large Stokes shift thanks to a rather efficient intramolecular photoinduced energy transfer process. Therefore, these new species could be used in the field of high-resolution light microscopy and found by engaging in bioimaging as well.

Aside from that, **GalTEBB-1** and **GalTEBB-2** showed good antibacterial properties that are ascribable to their structural features, and for these reasons, studies on their action against antibacterial biofilms are ongoing.

## 4. Experimental Compounds

Compound **6**: First, 2-propynil-*O*-*β*-galactopyranoside-2,3,4,6-tetraacetate (**5**) (0.43 g and 1.11 mmol, 1 equivalent), 1,4-bis-(2,3,4,6-tetra-*O*-acetyl-D-*β*-galactopyranosil)-2,5-diiodobenzene (**4**) (2.25 g and 2.20 mmol, 2 equivalent), Pd(PPh_3_)_4_ (26.48 mg and 0.023 mmol, 0.13 equivalent), and Et_3_N (10.6 mL) were dissolved into dry DMF (10.6 mL). The reaction mixture was maintained under stirring and an inert atmosphere until the disappearance of compound **5** (7 h) by TLC (hexane/ethyl acetate 55:45). The solvent was removed under reduced pressure, and the crude product was purified by a chromatographic column in silica gel, using as eluant hexane/ethyl acetate at a 70:30 ratio. Compound **6** was obtained as a low-melting solid (0.49 g, 0.39 mmol) (R*_f_* = 0.32, hexane/ethyl acetate 55:45) with a yield of 35%.

Compound **9**: Compound **BDP-R 7** (0.4 g, 0.89 mmol, 1 equivalent), 1,3,5-triethynylbenzene (1.07 g, 7.12 mmol, 8 equivalent), Pd(PPh_3_)_4_ (0.013 g, 0.11 mmol, 0.13 equivalent), and dry Et_3_N (12 mL) were dissolved in dry DMF (12 mL). The reaction mixture was maintained under stirring at room temperature and an inert atmosphere for 1 h [41]. The solvent was removed under reduced pressure, and the crude product was purified by column chromatography in silica gel using hexane/dichloromethane (75:25) → DCM 100% as an eluant, obtaining 0.34 g of the desired compound **9** as brilliant red powder (R*_f_* = 0.4 hexane/ethyl acetate (90:10)), with MP > 300 °C and a yield of 80%.

Compound **10**: The compound **BDP-G 8** (0.15 g, 0.21 mmol, 1 equivalent), compound **9** (0.30 g, 0.63 mmol, 3 equivalent) and Pd(PPh_3_)_4_ (0.032 g, 0.028 mmol, 0.13 equivalent) and dry Et_3_N (11.5 mL) were dissolved in dry DMF (11.5 mL) The reaction mixture was maintained under stirring at 60 °C and an inert atmosphere for 45 min. The solvent was removed under reduced pressure, and the crude product was purified by column chromatography in silica gel using hexane 100% → ethyl acetate 100% as an eluant, obtaining 0.17 g of the desired compound **10** (0.16 mmol) as dark green powder (R*_f_* = 0.2, hexane/ethyl acetate 75:25), with MP > 300 °C and a yield of 76%.

**GalTEBB-1**: Compound **10** (0.127 g, 0.12 mmol, 1 equivalent), compound **6** (0.15 g, 0.12 mmol, 1 equivalent), and Pd(PPh_3_)_4_ (0.018 g, 0.015 mmol, 0.13 equivalent), and dry Et_3_N (1.4 mL) were dissolved in dry DMF (1.4 mL). The reaction mixture was maintained under stirring at 65 °C and an inert atmosphere for 4 h. The solvent was removed under reduced pressure, and the crude product was purified by column chromatography in silica gel using dichloromethane/ethyl acetate (92:8) → DCM/ethyl acetate (85:15), obtaining 0.124 g of **GalTEBB-1** (0.056 mmol) as dark green powder (R*_f_* = 0.2, dichloromethane/ethyl acetate (80:20)), with MP = 225–227 °C and a yield of 47%.

**GalTEBB-2**: Iodomethane (1.4 mL, 22.5 mmol, 500 equivalent) and **GalTEBB-1** (0.10 g, 0.045 mmol, and 1 equivalent) were dissolved in DCM (3 mL). The reaction mixture was maintained under stirring at room temperature for 3 days. The reaction was monitored by TLC (acetonitrile/toluene (70:30)) until the disappearance of **GalTEBB-1**. **GalTEBB-2** was obtained (0.11 g 0.044 mmol), after evaporation as dark blue powder in an almost quantitative yield (Al_2_O_3_, R*_f_* = 0.34, acetonitrile/toluene (70:30)) with MP ˃ 300 °C.

## Figures and Tables

**Table 1:** Absorption and emission data in acetonitrile at 298 K.

	Absorption	Emission		
	*λ*_max_/nm (*ε*/M^−1^ cm^−1^)	*λ*_max_/nm	*τ*/ns	Φ
**GalTEBB-1**	500 (120,300) 700 (95,900)	790	2.2	0.20
**GalTEBB-2**	500 (102,600) 610 (122,300)	625	5.6	0.7

**Table 2:** Minimum inhibitory concentration (MIC) and minimum bactericidal concentration (MBC) of DMSO alone (%, *w*/*v*), GalTEBB-1 (µg/mL), and GalTEBB-2 (µg/mL) for *S. aureus* and *P. aeruginosa* at 37 °C.

Compounds	*P. aeruginosa*		*S. aureus*	
	MIC	MBC	MIC	MBC
DMSO alone	6.25%	12.5%	25%	25%
GalTEBB-1	125 µg/mL (6.25%)	125 µg/mL (6.25%)	250 µg/mL (12.5%)	250 µg/mL (12.5%)
GalTEBB-2	125 µg/mL (6.25%)	125 µg/mL (6.25%)	250 µg/mL (12.5%)	250 µg/mL (12.5%)

In parenthesis, we show the proportional value of DMSO in the galactoconjugate solutions (Table S1).

## Data Availability

Data are contained within the article and Supplementary Materials.

## Supplementary Materials

The following supporting information can be downloaded at: https://www.mdpi.com/article/10.3390/molecules29102299/s1, Figure S1: Excitation spectrum of **GalTEBB-1** in CH_3_CN solution registered @790 nm; Figure S2: Excitation spectrum of **GalTEBB-2** in CH_3_CN solution registered @690 nm; Figure S3: UV-Vis Absorption spectra of compound **GalTEBB-1** and **GalTEBB-2** in CH_3_CN; Figure S4: Emission spectra of compound **GalTEBB-1** and **GalTEBB-2** in CH_3_CN λ_ex_ 450 nm; Figure S5: Optical microscope images of cell line hFOB 1.19 in normal conditions (A) and exposed to **GalTEBB-1** (B) and 2 (C) at a final concentration of 62.5 µg/mL for 24 h; Figure S6: Antibacterial activity under a white-light source (26000 lux, fluence rate 3.81 mW/cm^2^) for 1 h (totalling 13.7 J/cm^2^ fluence) of DMSO (orange), **GalTEBB-1** (green) and **GalTEBB-2** (blue) against *P. aeruginosa* and *S. aureus*; Figure S7: ^1^H-NMR compound **6** in CDCl_3_; Figure S8: ^13^C-NMR compound **6** in CDCl_3_; Figure S9: ^1^H-NMR compound **9** in CDCl_3_; Figure S10: ^13^C-NMR compound **9** in CDCl_3_; Figure S11: ^1^H-NMR compound **10** in CDCl_3_; Figure S12: ^13^C-NMR compound **10** in CDCl_3_; Figure S13: ^1^H-NMR compound **GalTEBB-1** in CDCl_3_; Figure S14: ^13^C-NMR compound **GalTEBB-1** in CDCl_3_; Figure S15: ^1^H-NMR compound **GalTEBB-2** in acetone-d_6_; Figure S16: ^13^C-NMR compound **GalTEBB-2** in acetone-d_6_; Table S1: Correlation between of DMSO and bodipy-tagged galactoconjugates; Table S2: Bactericidal activity of DMSO, **GalTEBB-1** and **GalTEBB-2** against *P. aeruginosa* at different concentrations; Table S3: Bactericidal activity of DMSO, **GalTEBB-1** and **GalTEBB-2** against *S. aureus* at different concentrations. Refs. [32,42,43,44,45,46] are cited in Supplementary Materials.
