# Supplementary material for: Synthesis of Bodipy-Tagged Galactoconjugates and Evaluation of Their Antibacterial Properties
Source: Molecules. 2024 May 14;29(10):2299. doi: 10.3390/molecules29102299 (PMC11124175; doi:10.3390/molecules29102299)
Supplement: Supplementary file 1 [file molecules-29-02299-s001.zip › molecules-2985628-supplementary.pdf]

Synthesis of Bodipy-tagged galactoconjugates and evaluation of their antibacterial properties

Chiara Maria Antonietta Gangemi, Maura Monforte, Antonino Arrigo, Paola Maria Bonaccorsi, Sabrina Conoci, Antonella Iaconis, Fausto Puntoriero, Domenico Franco* and Anna Barattucci*

Dipartimento di Scienze Chimiche, Biologiche, Farmaceutiche ed Ambientali, Università degli Studi di Messina, V.le F. Stagno D'Alcontres 31, 98166 Messina, Italy

Electronic Supporting Information

Table of Contents

1.General Information	pg. S2
2.Experimental Section	pg. S3
3.Characterization Data	pg. S4
4.Fluorescence experiments	pg. S7
5.Biological data	pg. S8
6.NMR Spectra of products	pg. S12
7.References	pg. S22

1. General Information

Chemicals. Solvents were purified according to standard procedures. All the syntheses were monitored by TLC on commercially available precoated plates (silica gel 60 F_{254}), and the products were visualized with vanillin [1 g dissolved in MeOH (60 mL) and conc. H_2SO_4 (0.6 mL)] and UV lamp. Silica gel 60 was used for column chromatography.

Instrumentation. Proton (^1H) and carbon (^{13}C) NMR spectra were recorded on a Varian 500 spectrometer (at 500 MHz for ^1H; and 125 MHz for ^{13}C) using $CDCl_3$ or acetone-d_6 as solvents. Chemical shifts are given in parts per million (ppm) (δ relative to residual $CHCl_3$ peak (7.26 ppm and 77.0 ppm) or acetone peak (2.05 ppm and 30.5 ppm) for ^1H and ^{13}C), coupling constants (J) are given in Hertz, and the attributions are supported by heteronuclear single-quantum coherence (HSQC) and correlation spectroscopy (COSY) experiments. Mass analysis for final products were performed with a TSQ-Quantum access Triple Quadrupole Mass Spectrometer (Thermo Fisher Scientific, Waltham, MA, USA), equipped with a HESI (Heated ElectroSpray Ionization) source; analyses were run in positive mode. Mass spectrometer parameters: sheath gas flow rate, 30 (arbitrary units); aux gas flow rate, 15 (arbitrary units); spray voltage, 5.00 kV; capillary temperature, 250° C; tube lens voltage, 55 V; heater temperature, 270°C; scan mode: full scan.

UV/vis absorption spectra were recorded with a Jasco V-560 spectrophotometer. The luminescence was investigated with a Jobin Yvon-Spex Fluoromax P spectrofluorometer equipped with a Hamamatsu R3896 photomultiplier. The emission spectra were corrected for the photomultiplier response taking advantage of a program purchased with the instrument. Luminescence lifetimes were calculated on data registered with a Edinburgh OB 900 time-correlated single-photon-counting spectrometer and a Hamamatsu PLP 2 laser diode (59 ps pulse width at 408 nm) as excitation pulse. Emission quantum yields were determined by the optically diluted method, by using as luminescence quantum yield standards, $[Ru(bpy)_3]^{2+}$ (bpy = 2,2'-bipyridine),[3] and cresyl violet.[4]

Bacterial strains, media, and growth conditions. *Pseudomonas aeruginosa* (*P. aeruginosa*) ATCC27853 and *Staphylococcus aureus* (*S. aureus*) ATCC29213 were purchased from the American Type Culture Collection (LGC Promochem, Milan, Italy). Bacterial strains were chosen as representative of Gram-negative (*P. aeruginosa*) and -positive (*S. aureus*) pathogens associated with chronic infections.[5] *P. aeruginosa* was cultured in Miller´s Luria Broth (LB, Condalab, Madrid, Spain), while *S. aureus* in Trypticasein Soy Broth (TSB, Condalab, Madrid, Spain). Both bacterial strains were maintained in their respective media supplemented with 20% glycerol at -80 °C.

Antibacterial assay. *in vitro* evaluating antimicrobial activity was performed by broth microdilution assay of which a brief description follows. For each strain, semi-exponential broth culture was prepared at a final concentration of about 10^5 bacteria/mL starting from 0.5 McFarland inoculum (~1.5×10^8 bacteria/mL). Bacterial suspensions were dispensed in 50 mL tubes and added with several concentrations of **GalTEBB-1** and **GalTEBB-2** (15.6, 31.25, 62.5, 125 and 250 µg/mL). Then, 150 µL from each experimental condition were distributed in 96-well plates and incubated at 37 °C overnight (18 h) dissolved in dimethylsulfoxide (DMSO) cell culture grade (A3672, ITW Reagents, Monza, Italy). DMSO was also evaluated at 0.78, 1.56, 3.12, 6.25 and12.5% (w/v), representing proportional percentages present in the dilutions of **GalTEBB-1** and **GalTEBB-2** evaluated (**Table S1, ESI**). Bacterial growth under different experimental conditions was quantified by spectrophotometric reading at 540 nm (OD_{540}) with a microtiter plate reader (Multiskan GO, Thermo Scientific, Waltham, MA-USA) by using the following formula (equ. 1):

$$Normalized\ OD_{540} = \frac{OD_{540} - OD_{540}blank}{OD_{540}blank}$$

where OD_{540nm} blank is the experimental conditions without bacterial inoculum

Minimum inhibitory concentration (MIC) was evaluated by considering growth reduction over 90% with respect compared to the control condition without compounds (CTR+) using the following formula:

$$\text{bacterial growth } (\% = \left(\frac{normalized\ OD_{540}\ Condition}{normalized\ OD_{540}\ CTR+}\right) \times 100$$

MIC = *% bacterial inhibition* > 90%

Starting from MIC endpoints, minimum bactericidal concentration (MBC) was determined by subculturing to growth media added with 2% American Bacteriological Agar (Condalab, Madrid, Spain) and defined as the lowest concentration of compound resulting in microbial death.

Statistical analysis. All experimental conditions were analysed in triplicate as mean ± standard error (SE) and expressed as percentage versus control condition without compounds (CTR+). Differences among several conditions were evaluated by using one-way ANOVA analysis of variance followed by a Turkey post-hoc test for multiple comparisons. p-values lower than 0.05 were considered to be significant.

2. Experimental Section

1,4-Diiodo-2,5-dimethoxybenzene (1). A methanolic (20 mL) suspension of H_5IO_6 (2.9 g, 12.8 mmol) was kept under stirring at rt for 10′; I_2 (6.4 g, 25.2 mmol) was then added and, after further 10′ stirring, 2.70 g of 1,4-dimetossibenzene (19.5 mmol) were added. The mixture was warmed at 70°C and kept under stirring for 4h. After cooling, a solution of $Na_2S_2O_5$ (10% wt., 50 mL) was added and the obtained precipitate was filtered, washed with methanol and solubilized in DCM. The remaining precipitate was filtered, and evaporation of the mother liquors gave 6.8 g of 1 as white crystal. Yield 90%.

2,5-diiodo-idroquinone (2). 1,4-diiodo-2,5-dimetoxybenzene (**1**) (3.0 g, 7.7 mmol) was dissolved under argon atmosphere in 100 ml of dry DCM and the mixture was placed in an acetone/dry ice bath at -78°C. A solution of BBr_3 1,0 M in DCM (32.7 mmol) was added and the temperature was brought to rt, under continuous stirring for 16h. After this time, the solution was added to a water/ice mixture, with the formation of a white solid. After filtration, 1.99 g (5.5 mmol) of 2,5-diiodo-hydroquinone 2 were obtained. Yield 71%.

1,4-bis-O-trimethylsilyl-2,5-diiodo-idroquinone (3). To an anhydrous CH_3CN (6 mL) solution of **2** (1.0 g, 2.7 mmol), TMSCl (0.66 g, 6.07 mmol) and 1,1,1,3,3,3-hexamethyldisilazane (0.99 g, 6.13 mmol) were added. The reaction mixture was left under continuous stirring and in argon atmosphere for 16h. The solvent was removed under vacuum, and the obtained solid was added to hexane (10 mL), leaving the inorganic salts undissolved. The organic phase was washed twice with $NaHCO_3$ sat. and dried on Na_2SO_4. After solvent evaporation, 1.3 g (2.6 mmol) of **3** were obtained. Yield 94%.

1,4-bis-(2,3,4,6-tetra-O-acetyl-D-β-galactopyranosil)-2,5-diiodobenzene (4). To a solution of β-D-galactose pentaacetate (2.1 g, 5.30 mmol) in anhydrous DCM (25 mL) a solution of 1,4-bis-O trimetilsilyl-2,5-diiodo-idroquinone (**3**) (1.2 g, 2.4 mmol) in DCM (10 mL) and BF_3Et_2O (4.9 mL) were added. After 16h stirring at rt under argon atmosphere, the reaction was quenched with $NaHCO_3$ sat. The organic phase was dried over Na_2SO_4 and the solvent evaporated under vacuum. Compound **4** was obtained as pure white solid after crystallization from methanol of the crude product (1.4 g, 1.4 mmol). MP 183-185 °C. Yield 59%.

Compound 6

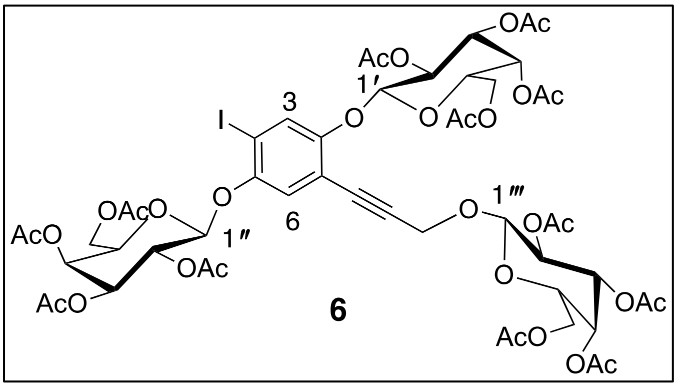

^{1}H NMR (500 MHz, $CDCl_3$) δ: 7.47 and 7.13 (2H, 2 x s, H-Ar), 5.57 and 5.51 (2H, 2 x dd, $J_{1,2}$= 8.0, $J_{2,3}$= 10.3, H-2’, H-2’’), 5.46-5.45 (2H, m, H-4’, H-4’’), 5.41-5.40 (1H, m, H-4’’’), 5.21 (1H, dd, $J_{1,2}$= 8.2, $J_{2,3}$= 10.3, H-2’’’), 5.14-5.10 (3H, m, H-3’, H-3’’, H-3’’’), 4.97 and 4.95 (2H, 2 x d, H-1’, H-1’’), 4.77 (1H, d, H-1’’’), 4.62 and 4.59 (2H, narrow AB system, J_{gem} =15.7, CH_2-C≡C), 4.31-4.02 (9H, m, H-5’, H-5’’, H-5’’’, H_2-6’, H_2-6’’, H2-6’’’), 2.20, 2.18, 2.16, 2.13, 2.11, 2.10, 2.05, 2.04, 2.03, 2.02 and 1.98 (36H, 11 x s, CH_3CO). ^{13}C NMR (125MHz, $CDCl_3$) δ: 170.3, 170.2, 170.1, 170.0, 169.9, 169.2 169.0, 162.4, 153.6, 151.5, 131.9, 131.8, 128.4, 128.3, 126.2, 120.0, 113.7, 100.3, 99.8, 99.2, 89.8, 87.8, 80.7, 71.3, 71.1, 70.6, 70.5, 70.4, 70.3, 68.7, 68.0, 67.9, 66.9, 66.8, 66.7, 61.7, 61.2, 61.1, 60.2, 57.0, 36.3, 31.2, 30.7, 21.1, 20.8, 20.7, 20.6, 20.5, 20.4, 20.3, 14.0. Anal. Calcd for $C_{51}H_{61}IO_{30}$ (1280,92): C, 47.82; H, 4.80; Found: C, 47.76; H, 4.80.

Compound 9

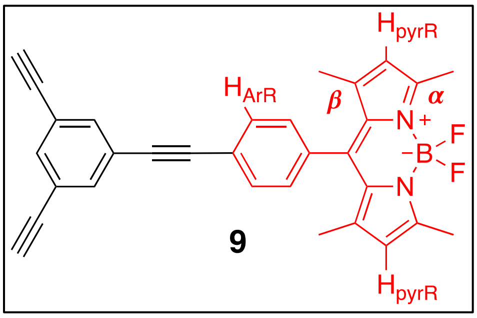

^{1}H NMR (500 MHz, $CDCl_3$): δ 7.65 (4H, m, H_{Ar}), 7.58 (1H, s, H_{Ar}), 7.29 (2H, d, J_{orto}=8.4, H_{Ar}), 5.99 (2H, s, H_{py}), 3.13 (2H, s, HC≡), 2.56 (s, 6H, 2xCH_3), 1.42 (s, 6H, 2xCH_3).^{13}C NMR (125 MHz, $CDCl_3$) δ: 155.8, 143.0, 140.6, 135.5, 135.4, 135.1, 132.4, 131.2, 128.3, 123.6, 123.4, 123.0, 121.4, 89.9, 88.7, 81.7, 78.7 and 14.6. Anal. Calcd for $C_{31}H_{23}BF_2N_2$ (472,35): C, 78.83; H, 4.91; N, 5.93; Found: C, 78.89; H, 4.92; N, 5.94.

Compound 10

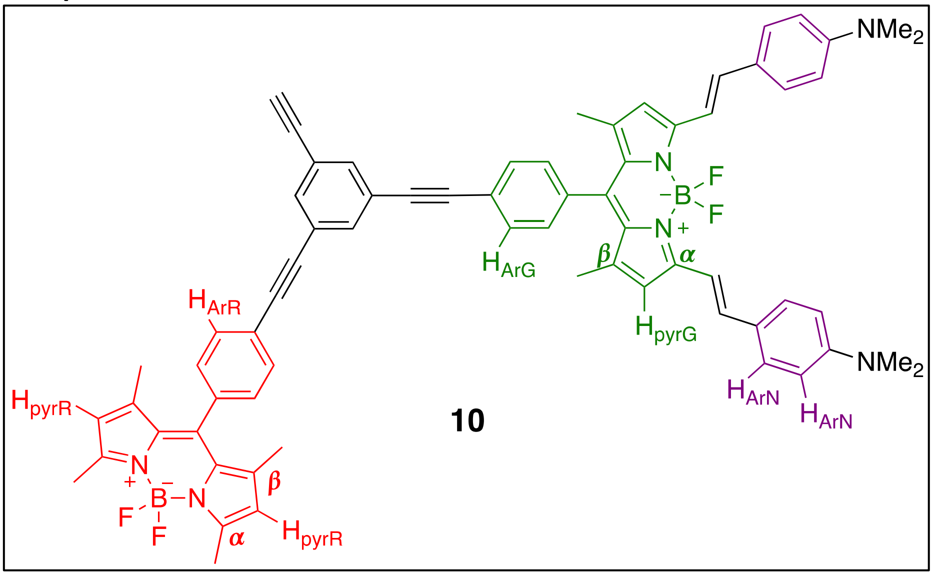

^{1}H NMR (500 MHz, $CDCl_3$) δ: 7.73-7.56 (12H, m, 3 x H_{Ar}, 2 x H_{ArG}, 4 x H_{ArN}, 2xH_{ArR} and HC=), 7.37 and 7.32 (4H, 2 x d, J_{orto}=7.8 and 7.9, 2 x H_{ArG}, 2 x H_{ArR}), 7.21 (2H, A part of an AB system, J_{vic} = 16.4, =CH), 6.72 (4H, d, J_{orto}=8.8, 4 x H_{ArN}), 6.61 (2H, s, H_{pyrG}), 6.00 (2H, s, H_{pyrR}), 3.16 (1H, s, HC≡), 3.04 (12H, s, 2 x $N(CH_3)_2$), 2.57 (6H, s, 2xCH_{3pyrR}), 1.48 and 1.44 (12H, 2 x s, 2xCH_{3pyrG}, 2xCH_{3pyrR}). ^{13}C NMR (125 MHz,

$CDCl_3$) δ: 155.8, 153.0, 150.9, 143.0, 140.6, 136.6, 136.3, 135.5, 134.9, 134.8, 134.6, 132.7, 132.4, 132.1, 131.2, 129.2, 129.1, 128.3, 125.1, 123.9, 123.7, 123.5, 123.1, 123.0, 121.4, 117.4, 114.9, 112.1, 90.3, 89.9, 88.9, 88.5, 81.8, 78.7, 40.3, 14.8, 14.6. Anal. Calcd for $C_{68}H_{58}B_2F_4N_6$ (1056,87): C, 77.28; H, 5.53; N, 7.95; Found: C, 76.98; H, 5.52; N, 7.93.

GalTEBB-1

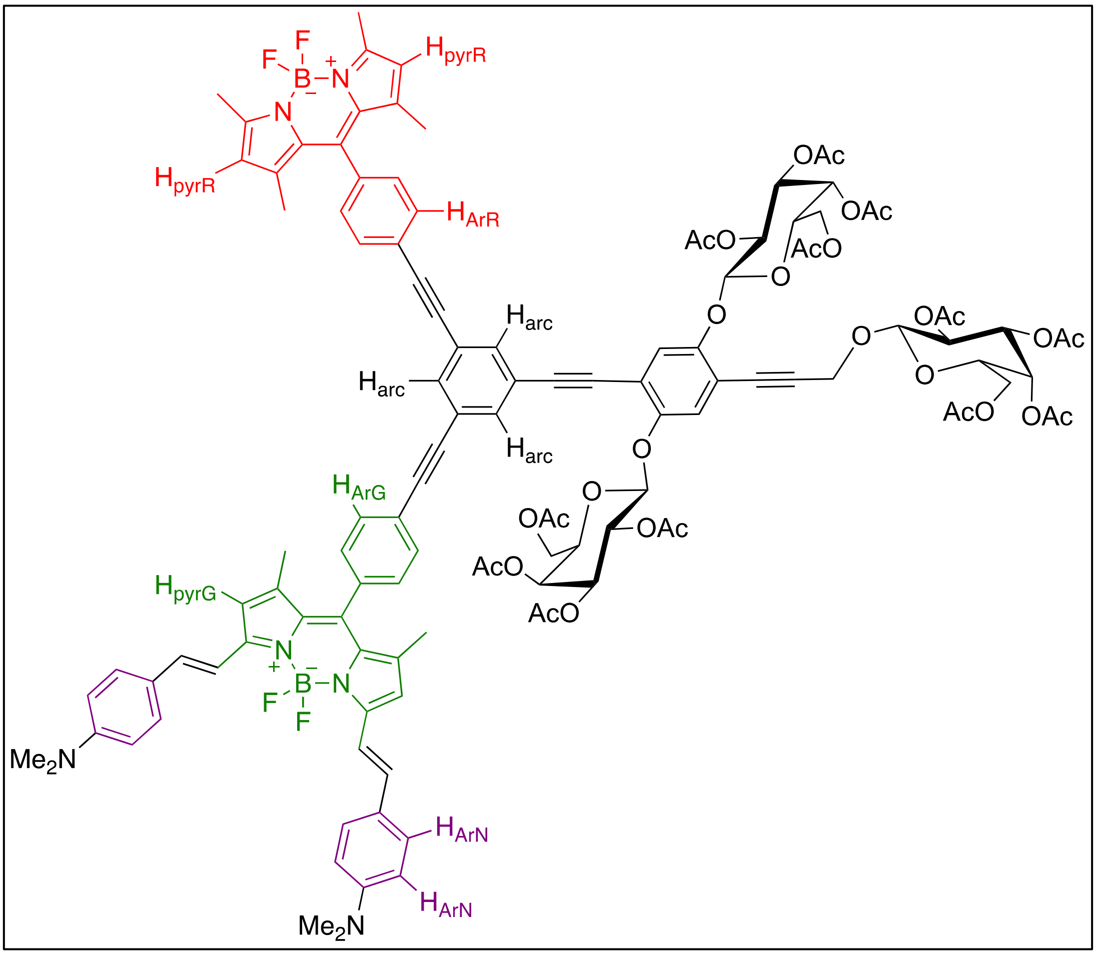

^1H NMR (500 MHz, $CDCl_3$) δ: 7.78, 7.76 and 7.74 (3H, 2 x t, J_m=1.5, H_{Arc}), 7.69 and 7.66 (4H, m, 2 x H_{ArG}, 2 x H_{ArR}), 7.56 and 7.20 (4H, AB system, J_{vic} =16.1, HC=CH), 7.53 (4H, d, J_o =8.8, 4 x H_{ArN}), 7.38 and 7.32 (4H, 2 x d, J_o =7.8, 2 x H_{ArG}, 2 x H_{ArR}),7.21 and 7.16 (2H, 2 x s, H-3,6), 6.71 (4H, d, 4 x H_{ArN}), 6.61 (2H, s, H_{pyrG}), 6.01 (2H, s, H_{pyrR}), 5.64 and 5.55 (2H, 2 x dd, $J_{1,2}$ = 7.8, $J_{2,3}$ = 10.8, H-2', H-2''), 5.49-5.47 (2H, m, H-4', H-4''), 5.41-5.40 (1H, m, H-4'''), 5.24 (1H, dd, $J_{1,2}$ = 7.8, $J_{2,3}$ = 10.8, H-2'''), 5.21-5.15 (3H, m, H-3', H-3'', H-3'''), 5.06 and 5.04 (2H, 2 x d, H-1', H-1''), 4.80 (1H, d, H-1'''), 4.66 and 4.62 (2H, narrow AB system, J_{gem} = 16.2, CH_2C=C), 4.32-4.04 (9H, m, H-5', H-5'', H-5''', H_2-6', H_2-6'', H_2-6'''), 3.03 (12H, s, 2 x $N(CH_3)_2$), 2.56 (6H, s, 2 x CH_{3pyrR}), 2.20, 2.19, 2.17, 2.10, 2.07, 2.06, 2.05, 2.04, 2.03, 2.02, 2.00, 1.98, 1.95 (36H, 13 x s, CH_3CO), 1.48 and 1.44 (12H, 2 x s, 2 x CH_{3pyrG}, 2 x CH_{3pyrR}). ^{13}C NMR (125 MHz, $CDCl_3$) δ: 170.5, 170.4, 170.2, 170.1, 170.6, 170.2, 169.4, 169.1, 155.8, 153.1, 153.0, 152.8, 150.9, 142.9, 140.6, 136.6, 136.3, 135.5, 135.0, 134.9, 134.7, 134.4, 134.3, 132.7, 132.4, 132.1, 131.1, 129.2, 129.1, 128.3, 125.0, 124.0, 123.8, 123.6, 123.5, 123.1, 121.4, 120.8, 119.8, 117.4, 115.1, 114.8, 114.5, 112.1, 100.4, 100.0, 99.4, 94.0, 90.8, 90.3, 90.0, 88.9, 88.6, 85.4, 81.1, 71.3, 71.2, 70.8, 70.7, 70.6, 70.5, 68.9, 68.3, 68.2, 67.1, 66.8, 61.5, 61.2, 60.3, 57.1, 53.4, 40.2, 20.8, 20.7, 20.6, 20.5, 14.8 and 14.6.

ESI (+)-MS m/z calcd for $C_{119}H_{118}B_2F_4N_6O_{30}$ [M+H+]=2209.8; found [M+H+]=2212.1 $C_{119}H_{116}D_2B_2F_4N_6O_{30}$ (please note that two protons exchanged with deuterium because of deuterated solvent). Anal. Calcd for C119H118B2F4N6O30 (2209,88): C, 64.68; H, 5.38; N, 3.80; Found: C, 64.58; H, 5.35; N, 3.80.

GalTEBB-2

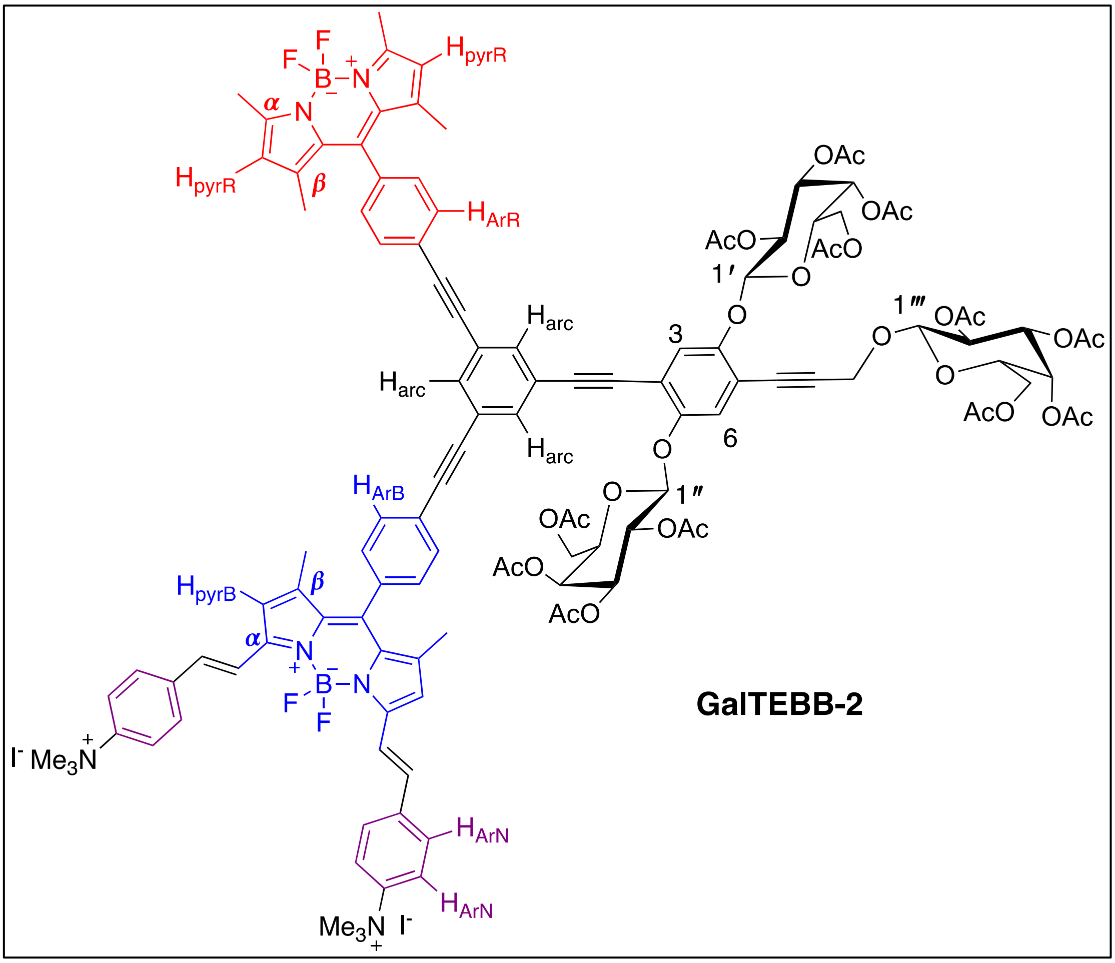

^{1}H NMR (500 MHz, acetone-d_6) δ: 8.34 (4H, d, J_o =8.8, 4 x H_{ArN}), 7.96-7.80 (12H, m, 2 x H_{ArB}, H_{Arc}, 4 x H_{ArN}, 2 x H_{ArR}, HC=C), 7.69 (2H, a part of an AB system, J_{vic} =16.1, C=CH), 7.63 and 7.54 (4H, 2 x d, J_o =7.9 and 7.8, 2 x H_{ArB}, 2 x H_{ArR}), 7.46 and 7.40 (2H, 2 x s, H-3,6), 7.01 (2H, s, H_{pyrB}), 6.15 (2H, s, H_{pyrR}), 5.63 - 5.41 and 5.35 - 5.15 (11H, m, H-2′, H-2′′, H-2′′′, H-4′, H-4′′, H-4′′′, H-1′, H-1′′), 4.98 (1H, d, $J_{1,2}$= 7.3, H-1′′′), 4.72 and 4.60 (2H, narrow AB system, J_{gem}= 15.5, CH_2C=C), 4.58-4.52 and 4.25-4.08 (9H, 2 x m, H-5′, H-5′′, H-5′′′, H_2-6′, H_2-6′′, H_2-6′′′), 4.01 (s, 18H, 2 x $N(CH_3)_3$), 2.52 (6H, s, 2 x CH_{3pyrR}), 2.20-1.93 (36H, 15 x s, CH_3CO), 1.60 and 1.49 (12H, 2 x s, 2 x CH_{3pyrB}, 2 x CH_{3pyrR}). ^{13}C NMR (125 MHz, acetone-d_6) δ: 171.5, 171.4, 171.3, 171.2, 170.9, 170.7, 170.6, 170.5, 157.1, 154.7, 154.3, 153.8, 148.8, 144.5, 142.8, 141.5, 140.2, 137.1, 136.8, 136.7, 136.6, 136.0, 135.9, 135.7, 135.0, 134.9, 134.2, 134.1, 132.5, 131.9, 131.8, 130.6, 130.1, 125.7, 125.6, 125.3, 125.0, 123.1, 122.9, 121.4, 120.4, 116.1, 115.7, 101.1, 100.9, 94.8, 92.9, 92.1, 91.7, 90.3, 90.0, 87.6, 82.3, 73.0, 72.9, 72.4, 72.3, 72.1, 70.4, 70.0, 69.9, 69.0, 68.9, 63.2, 62.8, 58.7, 58.2, 21.6, 21.5, 21.3, 21.2, 15.8, 15.4 and 15.3. ESI (+)-MS m/z calcd for $C_{121}H_{124}B_2F_4N_6O_{30}^{2+}$; [$M^{2+}$]=1119.93 found [$M^{2+}$]=1123.82 $C_{121}H_{116}D_8B_2F_4N_6O_{30}^{2+}$ (please note that eight protons exchanged with deuterium because of deuterated solvent). Anal. Calcd for $C_{121}H_{124}B_2F_4I_2N_6O_{30}$ (2493,76): C, 58.28; H, 5.01; N, 3.37; Found: C, 58.19; H, 5.04; N, 3.36.

3. Fluorescence experiments

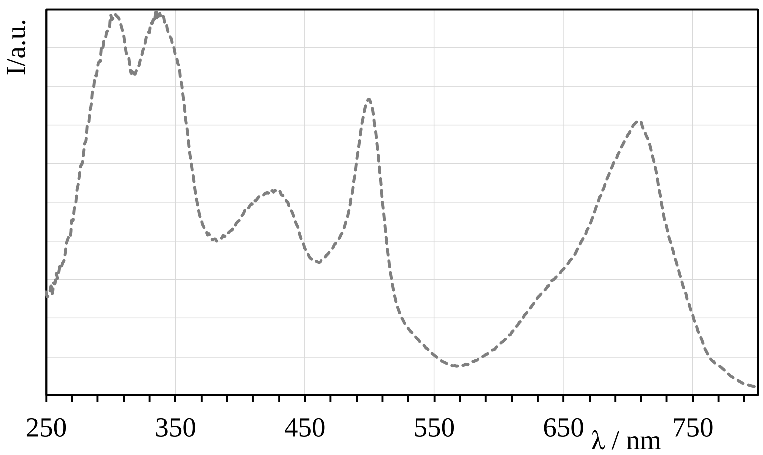

<u>Figure S1.</u> Excitation spectrum of **GalTEBB-1** in CH_3CN solution registered @790 nm.

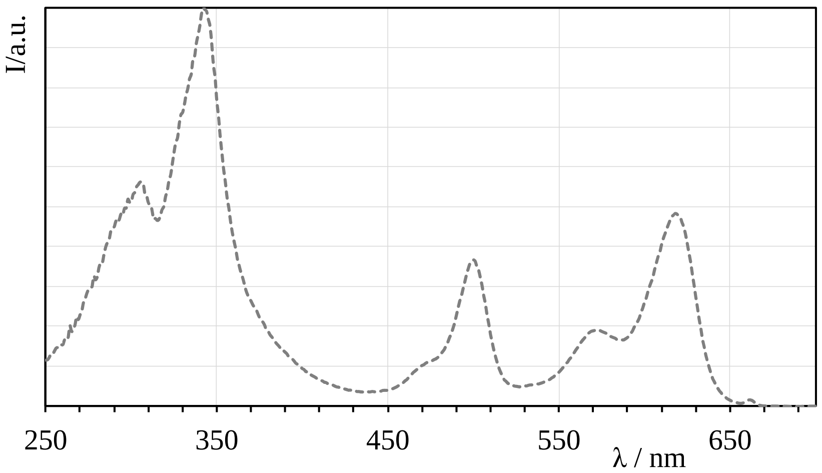

<u>Figure S2.</u> Excitation spectrum of **GalTEBB-2** in CH_3CN solution registered @690 nm.

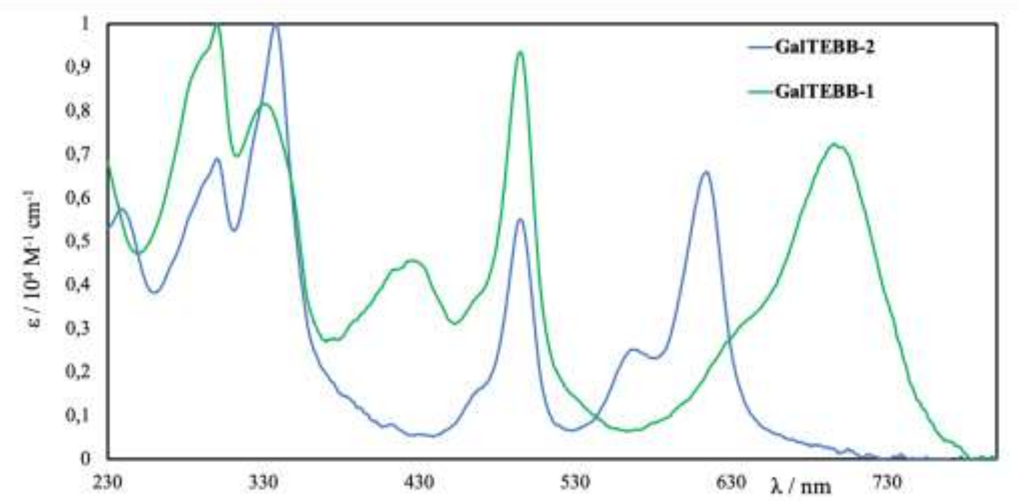

<u>Figure S3</u>. UV-Vis Absorption spectra of compound **GalTEBB-1** and **GalTEBB-2** in CH_3CN.

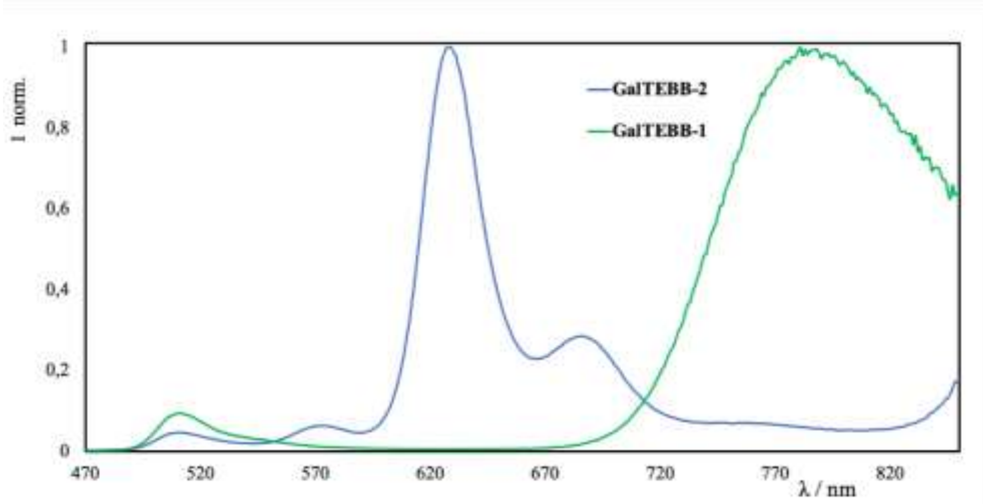

Figure S4. Emission spectra of compound **GalTEBB-1** and **GalTEBB-2** in CH_3CN λ_{ex} 450 nm.

4. Biological data

Table S1. Correlation between of DMSO and bodipy-tagged galactoconjugates

DMSO (%)	*GalTEBB (µg/mL)*
12.5	250
6.25	125
3.12	62.5
1.56	31.25
0.78	15.6

Table S2. Bactericidal activity of DMSO, **GalTEBB-1** and **GalTEBB-2** against *P. aeruginosa* at different concentrations

12.5 % DMSO - 250 µg/mL GalTEBB					
**Tukey's multiple comparisons test**	**Mean Diff,**	**95,00% CI of diff,**	**Significant?**	**Summary**	**Adjusted P Value**
**CTR vs. GalTEBB-1**	7,155	7,093 to 7,217	Yes	***	<0,001
**CTR vs. GalTEBB-2**	7,171	7,109 to 7,233	Yes	***	<0,001
**CTR vs. DMSO**	7,198	7,136 to 7,260	Yes	***	<0,001
**GalTEBB-1 vs. GalTEBB-2**	0,01667	-0,04517 to 0,07850	No	ns	0,82
**GalTEBB-1 vs. DMSO**	0,04333	-0,01850 to 0,1052	No	ns	0,19
**GalTEBB-2 vs. DMSO**	0,02667	-0,03517 to 0,08850	No	ns	0,54
6.25 % DMSO - 125 µg/mL GalTEBB					
**Tukey's multiple comparisons test**	**Mean Diff,**	**95,00% CI of diff,**	**Significant?**	**Summary**	**Adjusted P Value**
**CTR vs. GalTEBB-1**	7,151	7,102 to 7,200	Yes	***	<0,001
**CTR vs. GalTEBB-2**	7,161	7,112 to 7,210	Yes	***	<0,001
**CTR vs. DMSO**	7,078	7,029 to 7,127	Yes	***	<0,001
**GalTEBB-1 vs. GalTEBB-2**	0,01000	-0,03898 to 0,05898	No	ns	0,91
**GalTEBB-1 vs. DMSO**	-0,07333	-0,1223 to -0,02435	Yes	**	0,006
**GalTEBB-2 vs. DMSO**	-0,08333	-0,1323 to -0,03435	Yes	**	0,003
3.12 % DMSO - 62.5 µg/mL GalTEBB					

Tukey's multiple comparisons test	Mean Diff,	95,00% CI of diff,	Significant?	Summary	Adjusted P Value
**CTR vs. GalTEBB-1**	5,544	4,822 to 6,266	Yes	***	<0,001
**CTR vs. GalTEBB-2**	5,236	4,515 to 5,958	Yes	***	<0,001
**CTR vs. DMSO**	2,925	2,203 to 3,646	Yes	***	<0,001
**GalTEBB-1 vs. GalTEBB-2**	-0,3077	-1,029 to 0,4140	No	ns	0,55
**GalTEBB-1 vs. DMSO**	-2,619	-3,341 to -1,898	Yes	***	<0,001
**GalTEBB-2 vs. DMSO**	-2,312	-3,033 to -1,590	Yes	***	<0,001
1.56 % DMSO - 31.25 µg/mL GalTEBB					
**Tukey's multiple comparisons test**	**Mean Diff,**	**95,00% CI of diff,**	**Significant?**	**Summary**	**Adjusted P Value**
**CTR vs. GalTEBB-1**	4,061	3,280 to 4,842	Yes	***	<0,001
**CTR vs. GalTEBB-2**	3,479	2,698 to 4,260	Yes	***	<0,001
**CTR vs. DMSO**	1,968	1,187 to 2,749	Yes	***	<0,001
**GalTEBB-1 vs. GalTEBB-2**	-0,5827	-1,364 to 0,1983	No	ns	0,16
**GalTEBB-1 vs. DMSO**	-2,093	-2,874 to -1,312	Yes	***	<0,001
**GalTEBB-2 vs. DMSO**	-1,511	-2,292 to -0,7297	Yes	**	0,001
0.78 % DMSO - 15.6 µg/mL GalTEBB					
**Tukey's multiple comparisons test**	**Mean Diff,**	**95,00% CI of diff,**	**Significant?**	**Summary**	**Adjusted P Value**
**CTR vs. GalTEBB-1**	3,061	2,438 to 3,684	Yes	***	<0,001
**CTR vs. GalTEBB-2**	2,443	1,820 to 3,066	Yes	***	<0,001
**CTR vs. DMSO**	1,845	1,222 to 2,468	Yes	***	<0,001
**GalTEBB-1 vs. GalTEBB-2**	-0,6180	-1,241 to 0,004938	No	ns	0,05
**GalTEBB-1 vs. DMSO**	-1,216	-1,839 to -0,5934	Yes	**	0,001
**GalTEBB-2 vs. DMSO**	-0,5983	-1,221 to 0,02460	No	ns	0,06

<u>*Table S3*</u>. Bactericidal activity of DMSO, **GalTEBB-1** and **GalTEBB-2** against *S. aureus* at different concentrations

12.5 % DMSO - 250 µg/mL GalTEBB					
**Tukey's multiple comparisons test**	**Mean Diff,**	**95,00% CI of diff,**	**Significant?**	**Summary**	**Adjusted P Value**
**CTR vs. GalTEBB-1**	5,174	5,010 to 5,338	Yes	***	<0,001
**CTR vs. GalTEBB-2**	5,174	5,010 to 5,338	Yes	***	<0,001
**CTR vs. DMSO**	4,430	4,266 to 4,594	Yes	***	<0,001
**GalTEBB-1 vs. GalTEBB-2**	0,000	-0,1640 to 0,1640	No	ns	>0,99
**GalTEBB-1 vs. DMSO**	-0,7433	-0,9074 to -0,5793	Yes	***	<0,001
**GalTEBB-2 vs. DMSO**	-0,7433	-0,9074 to -0,5793	Yes	***	<0,001
6.25 % DMSO - 125 µg/mL GalTEBB					
**Tukey's multiple comparisons test**	**Mean Diff,**	**95,00% CI of diff,**	**Significant?**	**Summary**	**Adjusted P Value**
**CTR vs. GalTEBB-1**	4,397	4,043 to 4,751	Yes	***	<0,001
**CTR vs. GalTEBB-2**	4,000	3,646 to 4,354	Yes	***	<0,001
**CTR vs. DMSO**	1,483	1,129 to 1,837	Yes	***	<0,001
**GalTEBB-1 vs. GalTEBB-2**	-0,3967	-0,7506 to -0,04271	Yes	*	0,03
**GalTEBB-1 vs. DMSO**	-2,914	-3,268 to -2,560	Yes	***	<0,001
**GalTEBB-2 vs. DMSO**	-2,518	-2,872 to -2,164	Yes	***	<0,001

3.12 % DMSO - 62.5 µg/mL GalTEBB					
**Tukey's multiple comparisons test**	**Mean Diff,**	**95,00% CI of diff,**	**Significant?**	**Summary**	**Adjusted P Value**
**CTR vs. GalTEBB-1**	3,937	3,559 to 4,315	Yes	***	<0,001
**CTR vs. GalTEBB-2**	3,439	3,061 to 3,817	Yes	***	<0,001
**CTR vs. DMSO**	1,165	0,7870 to 1,543	Yes	***	<0,001
**GalTEBB-1 vs. GalTEBB-2**	-0,4980	-0,8760 to -0,1200	Yes	*	0,01
**GalTEBB-1 vs. DMSO**	-2,772	-3,150 to -2,394	Yes	***	<0,001
**GalTEBB-2 vs. DMSO**	-2,274	-2,652 to -1,896	Yes	***	<0,001
1.56 % DMSO - 31.25 µg/mL GalTEBB					
**Tukey's multiple comparisons test**	**Mean Diff,**	**95,00% CI of diff,**	**Significant?**	**Summary**	**Adjusted P Value**
**CTR vs. GalTEBB-1**	3,397	2,982 to 3,812	Yes	***	<0,001
**CTR vs. GalTEBB-2**	2,650	2,236 to 3,065	Yes	***	<0,001
**CTR vs. DMSO**	0,9217	0,5071 to 1,336	Yes	***	<0,001
**GalTEBB-1 vs. GalTEBB-2**	-0,7467	-1,161 to -0,3321	Yes	**	0,002
**GalTEBB-1 vs. DMSO**	-2,475	-2,890 to -2,061	Yes	***	<0,001
**GalTEBB-2 vs. DMSO**	-1,729	-2,143 to -1,314	Yes	***	<0,001
0.78 % DMSO - 15.6 µg/mL GalTEBB					
**Tukey's multiple comparisons test**	**Mean Diff,**	**95,00% CI of diff,**	**Significant?**	**Summary**	**Adjusted P Value**
**CTR vs. GalTEBB-1**	2,304	1,969 to 2,639	Yes	***	<0,001
**CTR vs. GalTEBB-2**	2,010	1,675 to 2,345	Yes	***	<0,001
**CTR vs. DMSO**	0,7850	0,4501 to 1,120	Yes	***	<0,001
**GalTEBB-1 vs. GalTEBB-2**	-0,2933	-0,6282 to 0,04157	No	ns	0,09
**GalTEBB-1 vs. DMSO**	-1,519	-1,854 to -1,184	Yes	***	<0,001
**GalTEBB-2 vs. DMSO**	-1,225	-1,560 to -0,8904	Yes	***	<0,001

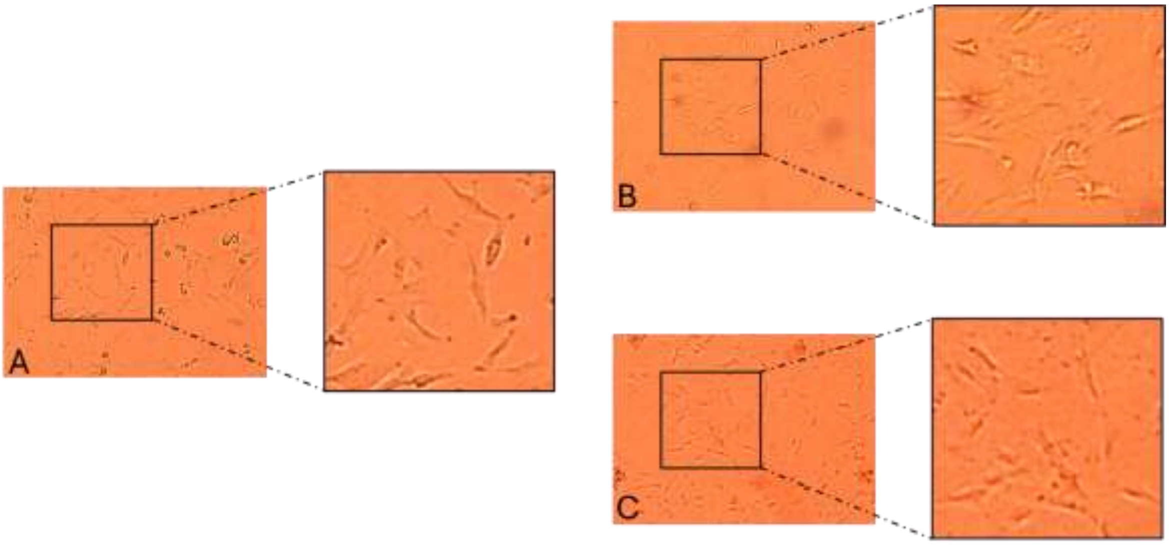

Figure S5 Optical microscope images of cell line hFOB 1.19 in normal conditions (A) and exposed to GalTEBB-1 (B) and 2 (C) at a final concentration of 62.5µg/mL for 24h.

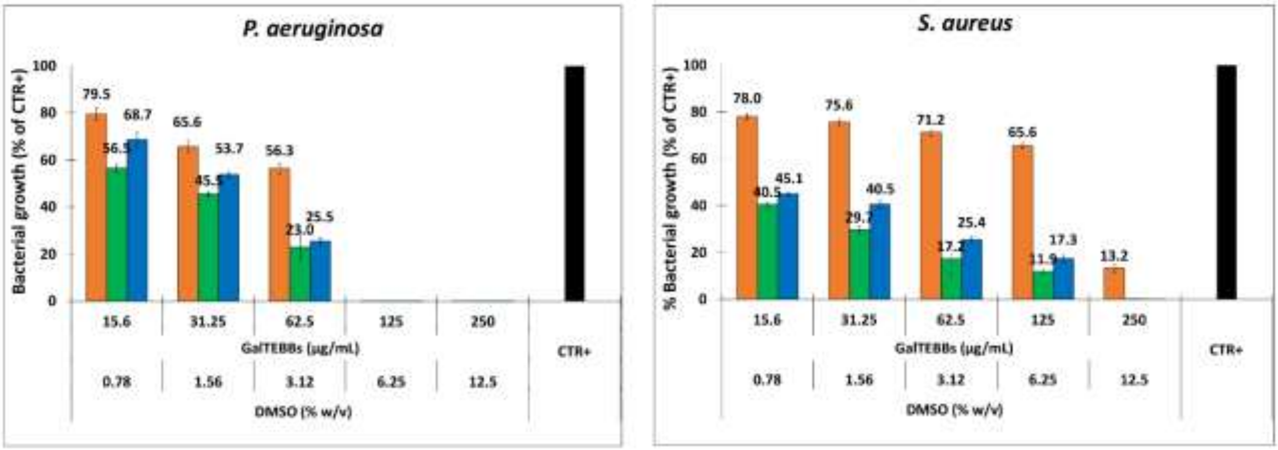

Figure S6: Antibacterial activity under a white-light source (26000 lux, fluence rate 3.81 mW/cm^2) for 1 h (totalling 13.7 J/cm^2 fluence) of DMSO (orange), GalTEBB-1 (green) and GalTEBB-2 (blue) against *P. aeruginosa* and *S. aureus.*

5. NMR Spectra of products

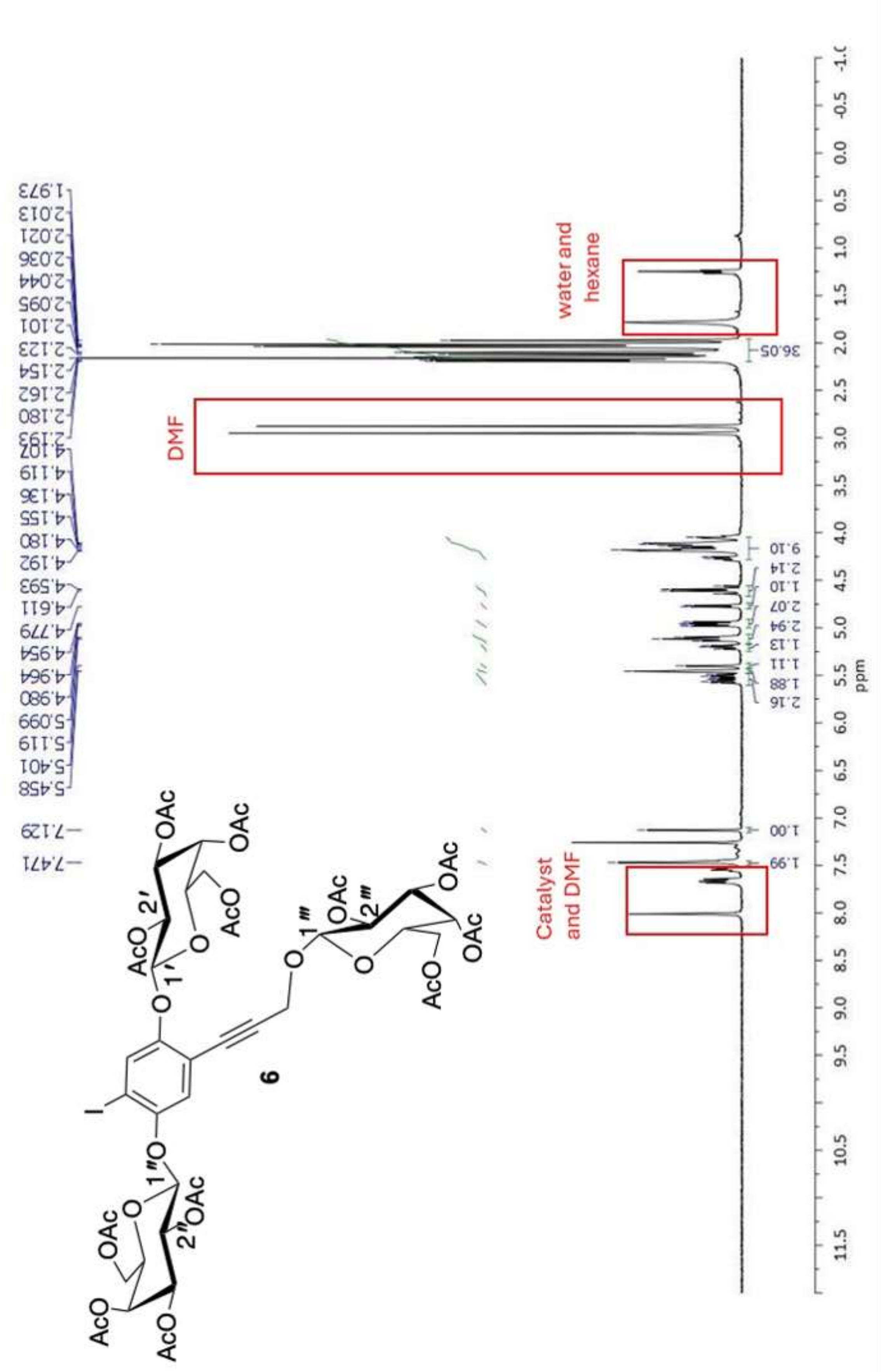

Figure S7. ^{1}H-NMR compound **6** in $CDCl_3$

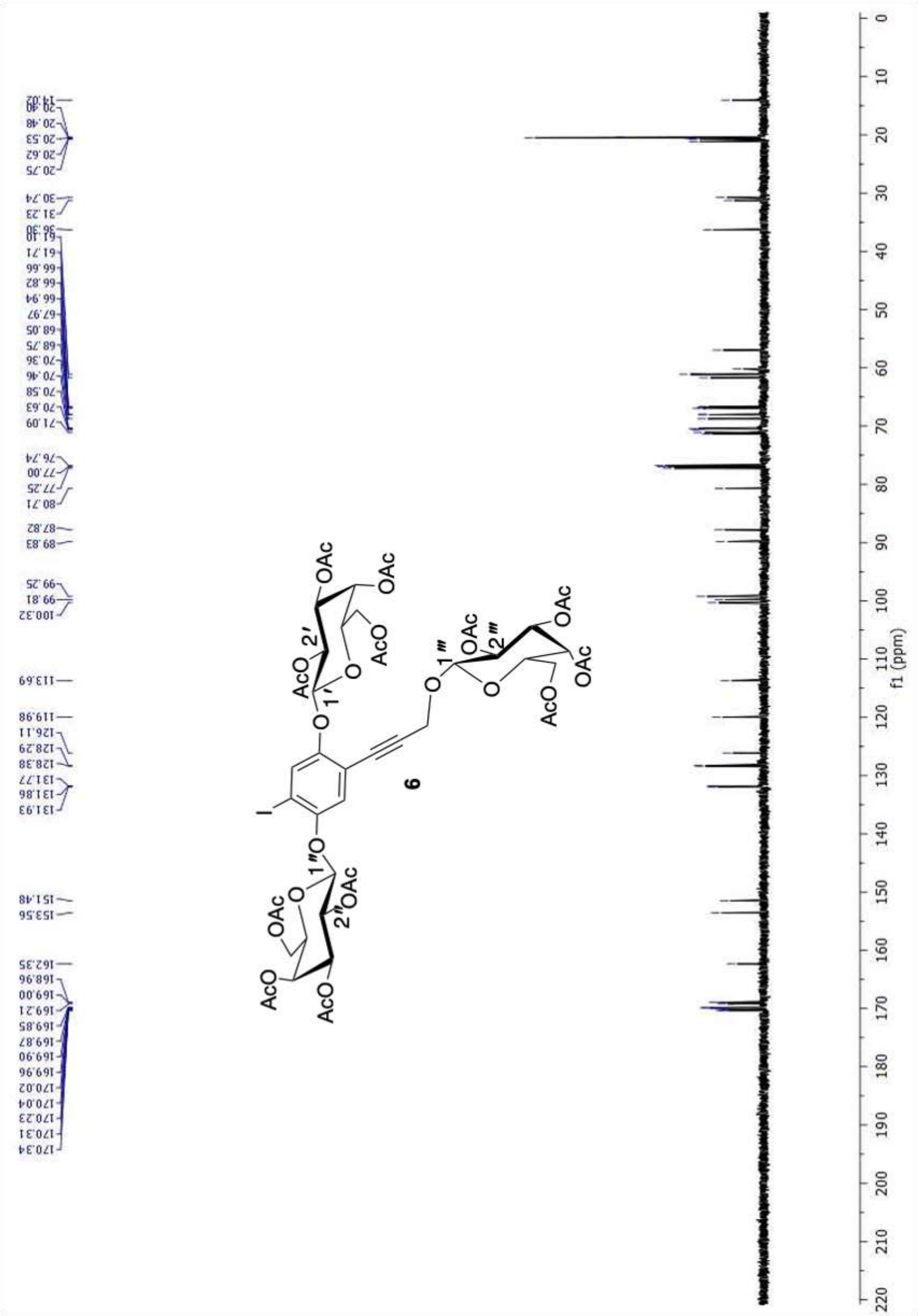

Figure S8. ^{13}C-NMR compound **6** in $CDCl_3$

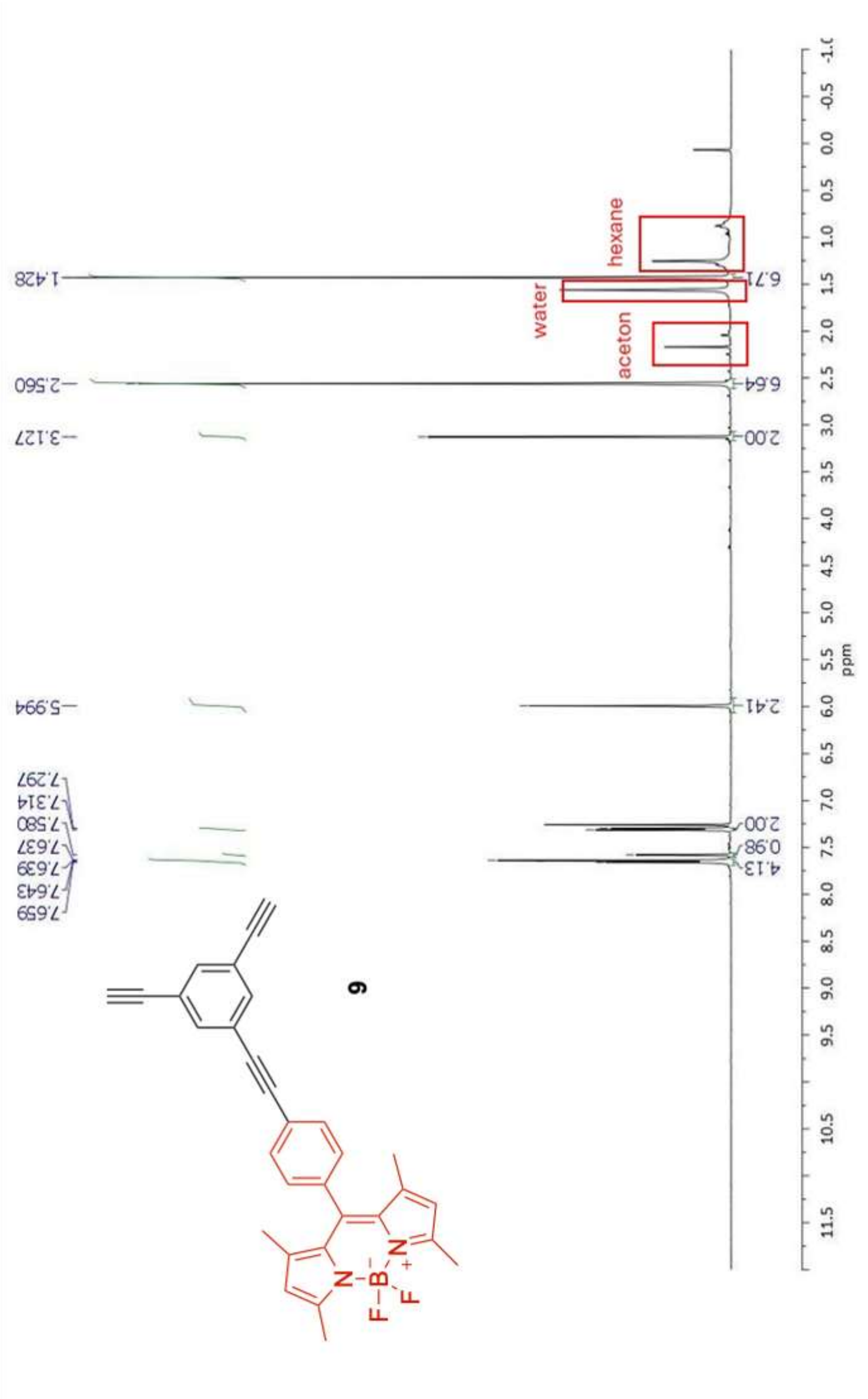

Figure S9. ^{1}H-NMR compound **9** in $CDCl_3$

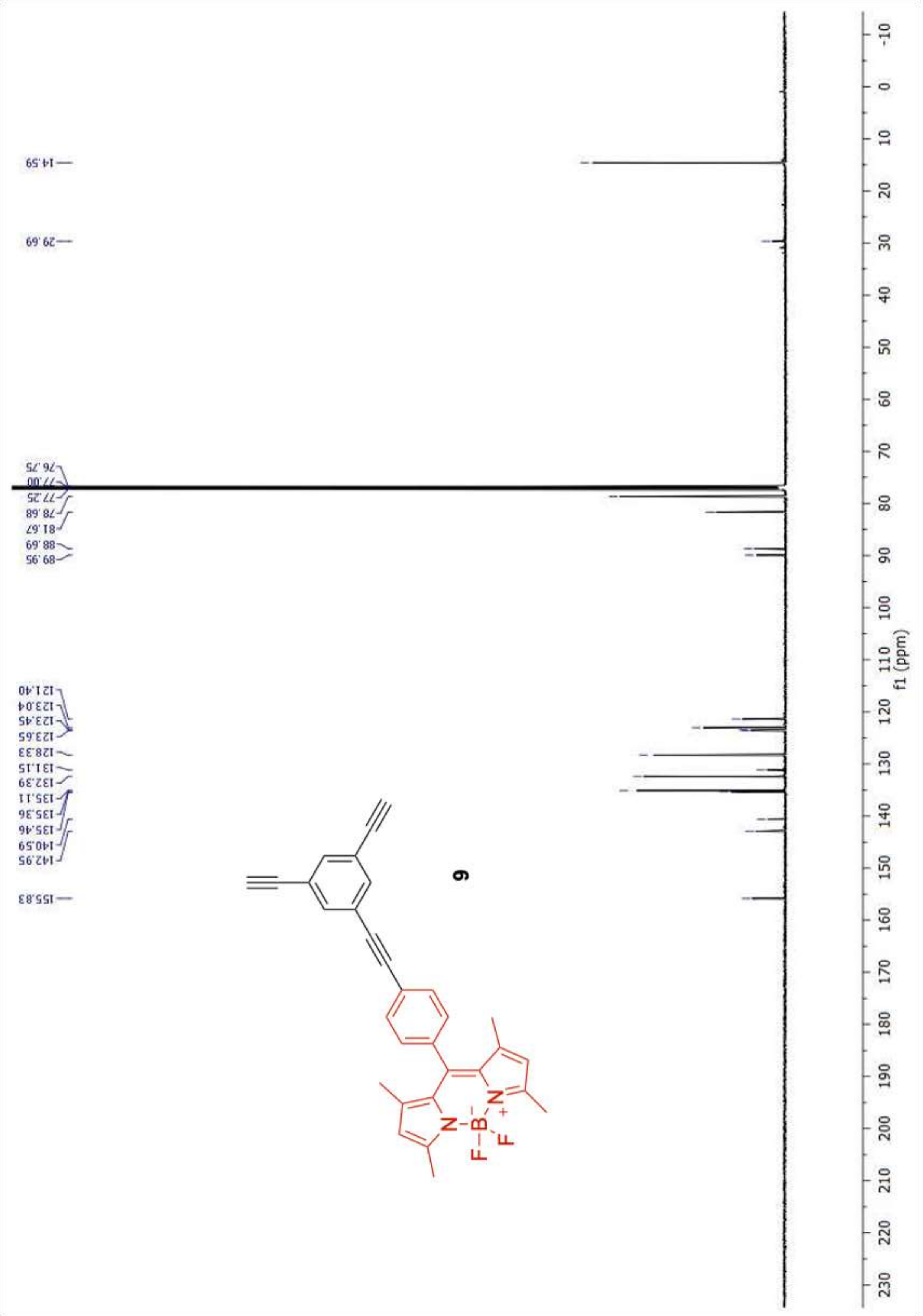

<u>Figure S10</u>. ^{13}C-NMR compound **9** in $CDCl_3$

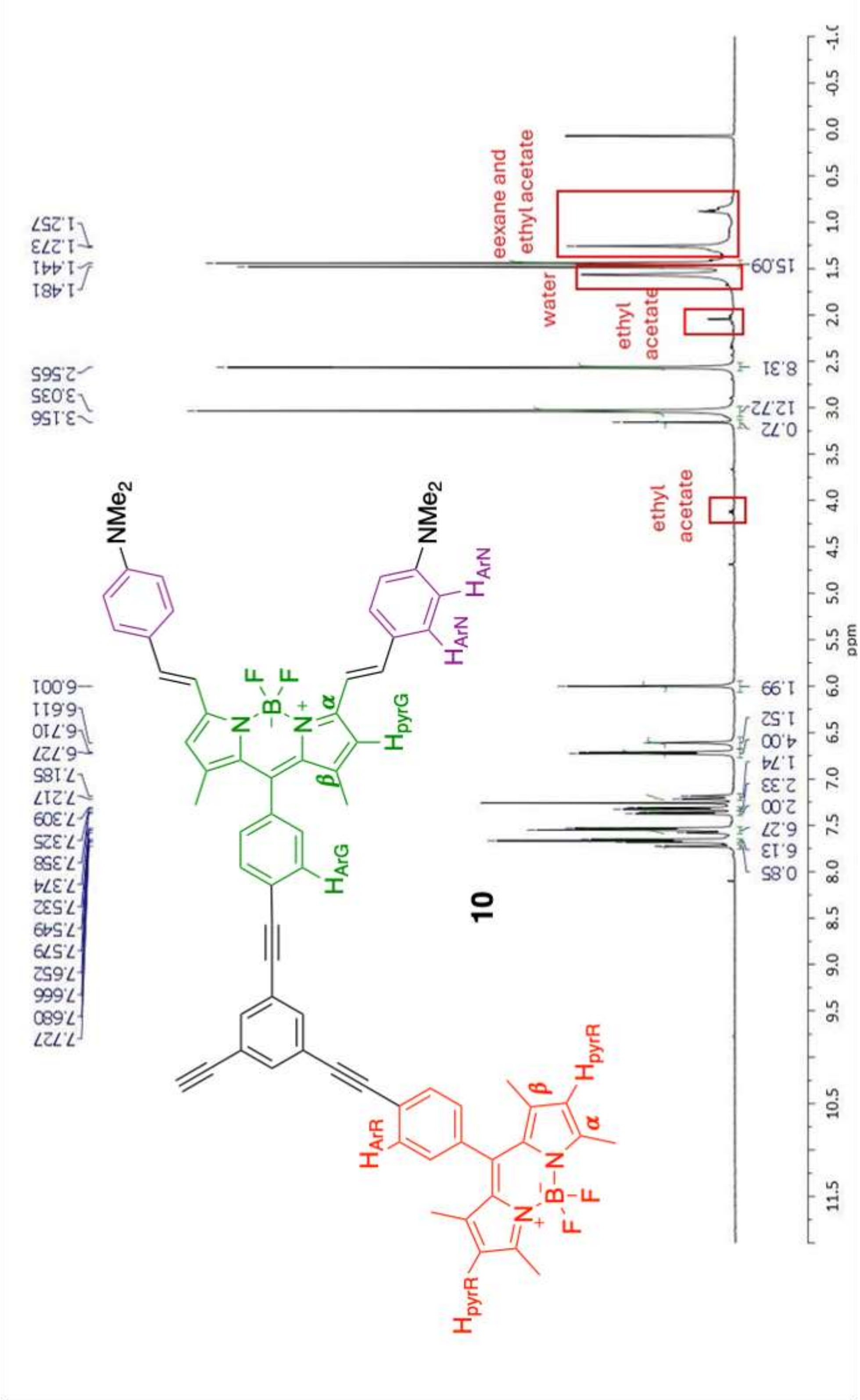

<u>Figure S11</u>. ^{1}H-NMR compound **10** in $CDCl_3$

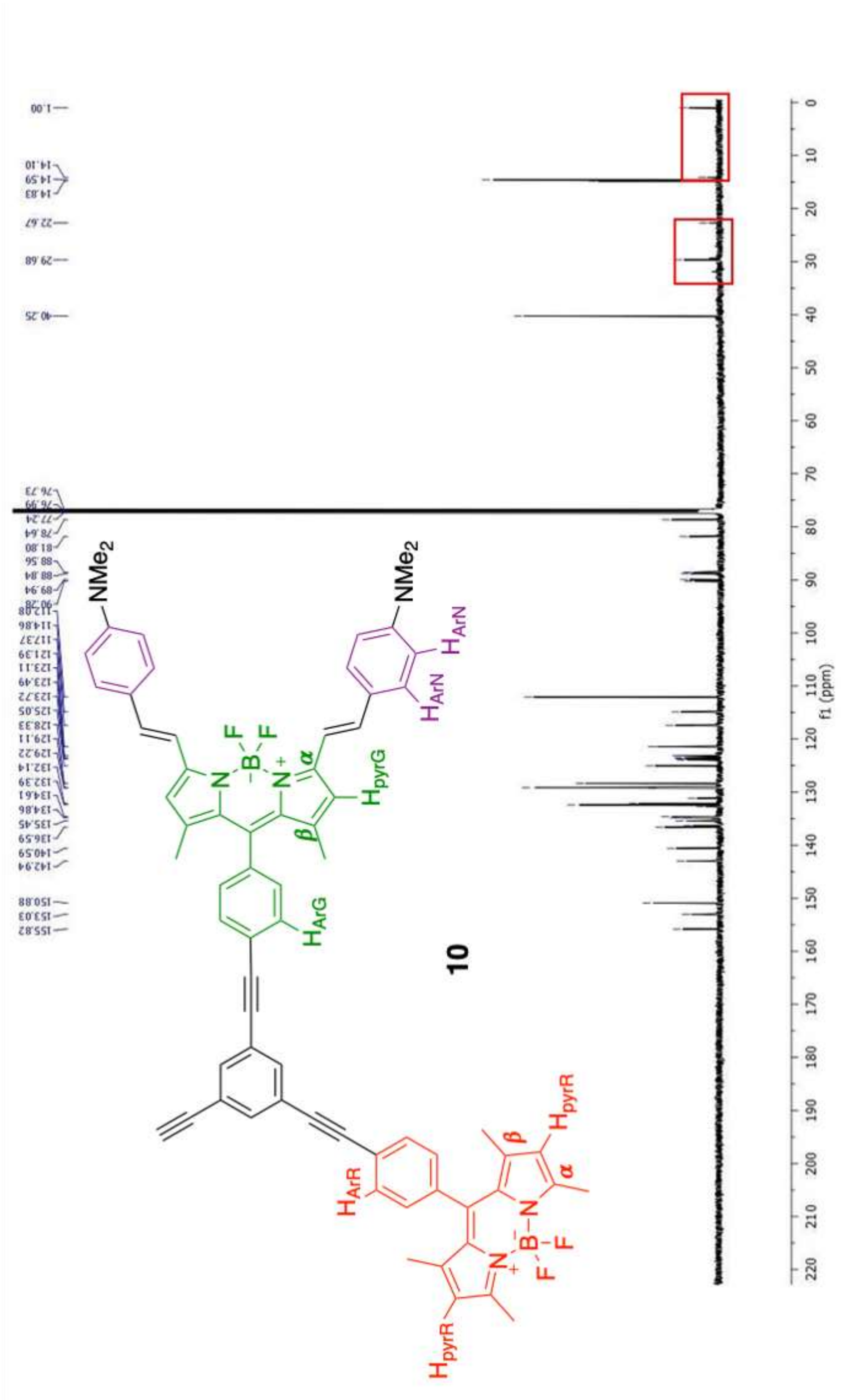

Figure S12. ^{13}C-NMR compound **10** in $CDCl_3$

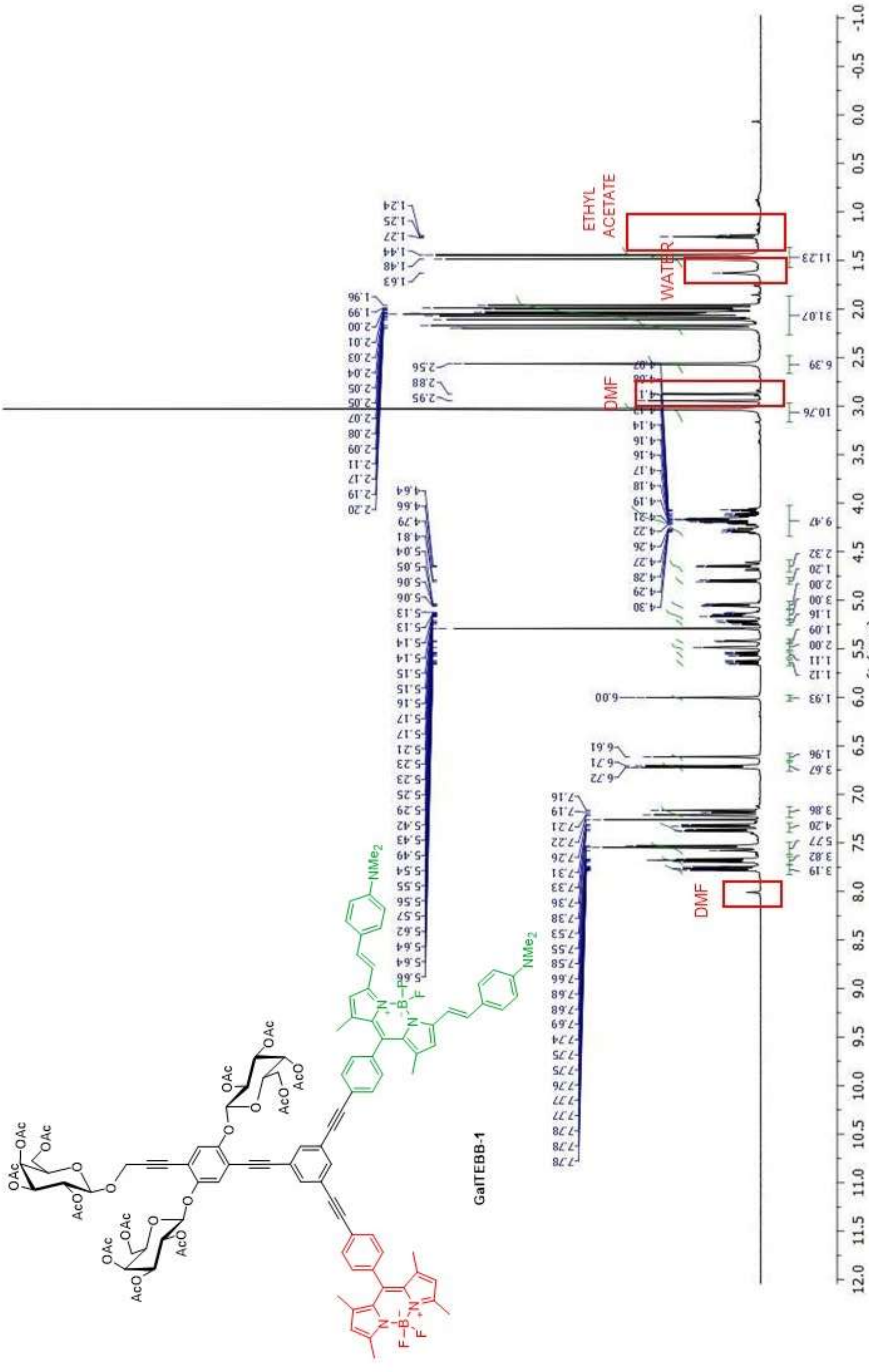

<u>Figure S13.</u> ^{1}H-NMR compound **GalTEBB-1** in $CDCl_3$

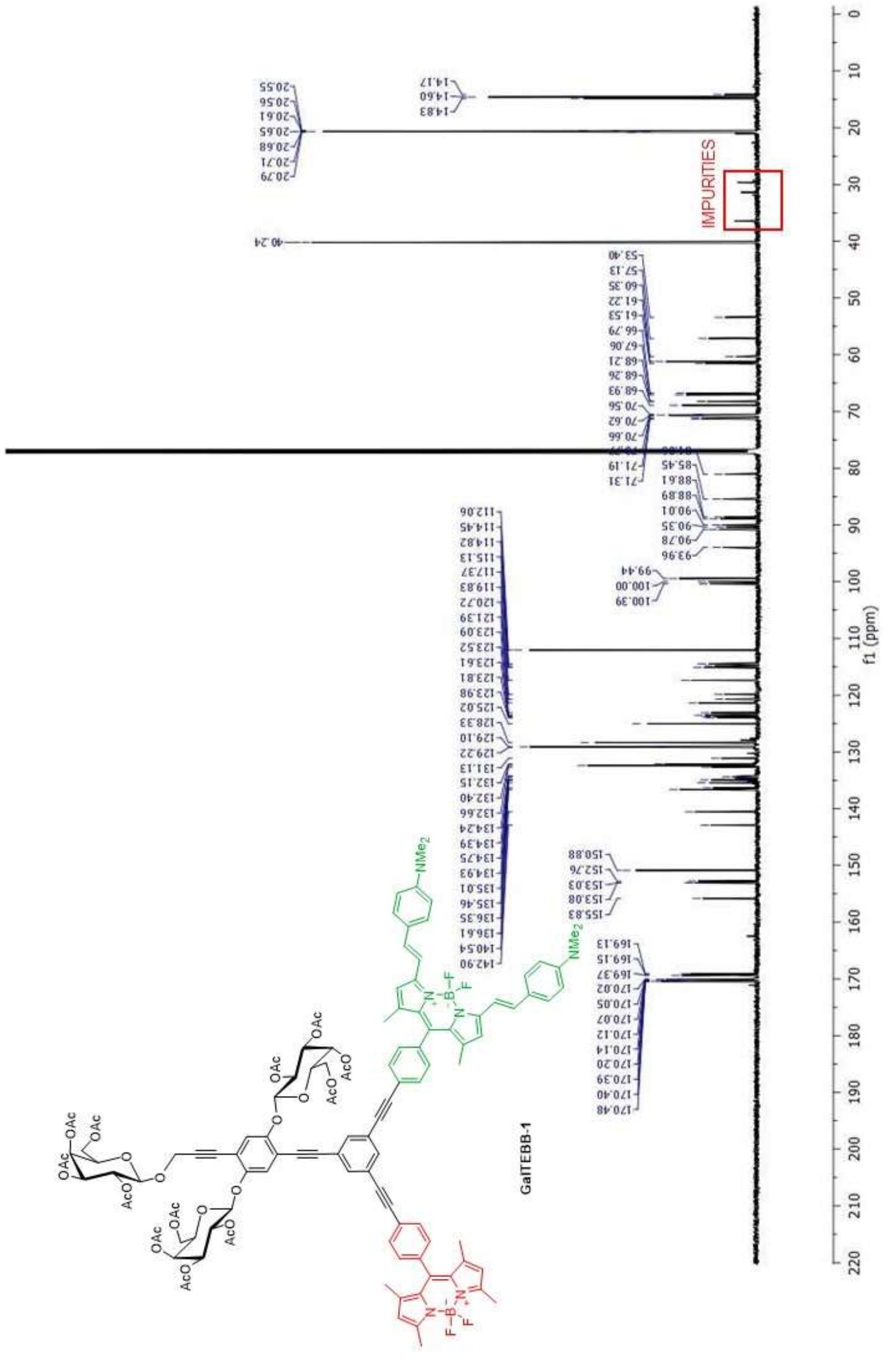

__Figure S14.__ ^{13}C-NMR compound **GalTEBB-1** in $CDCl_3$

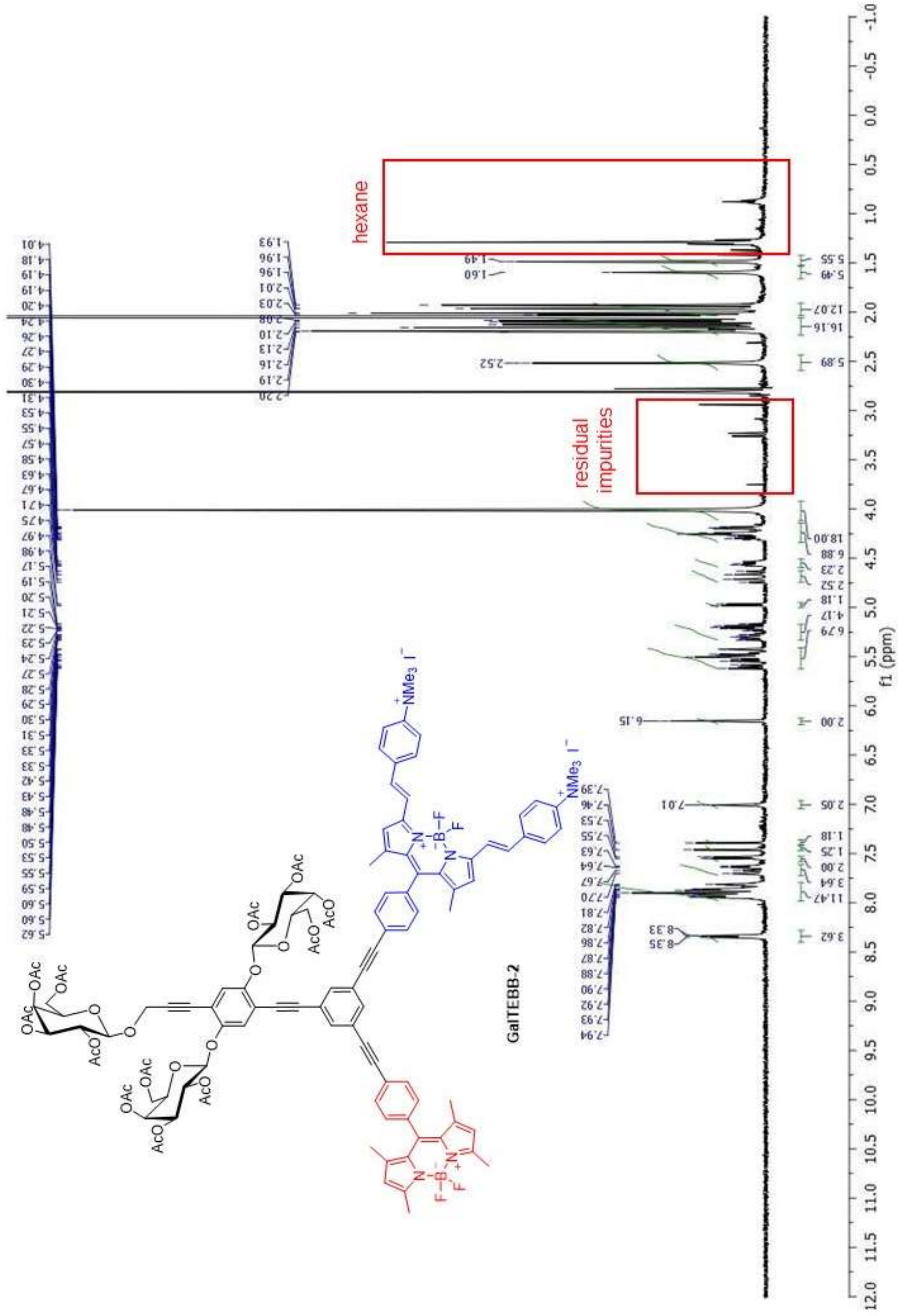

Figure S15. ^{1}H-NMR compound **GalTEBB-2** in acetone-d_6

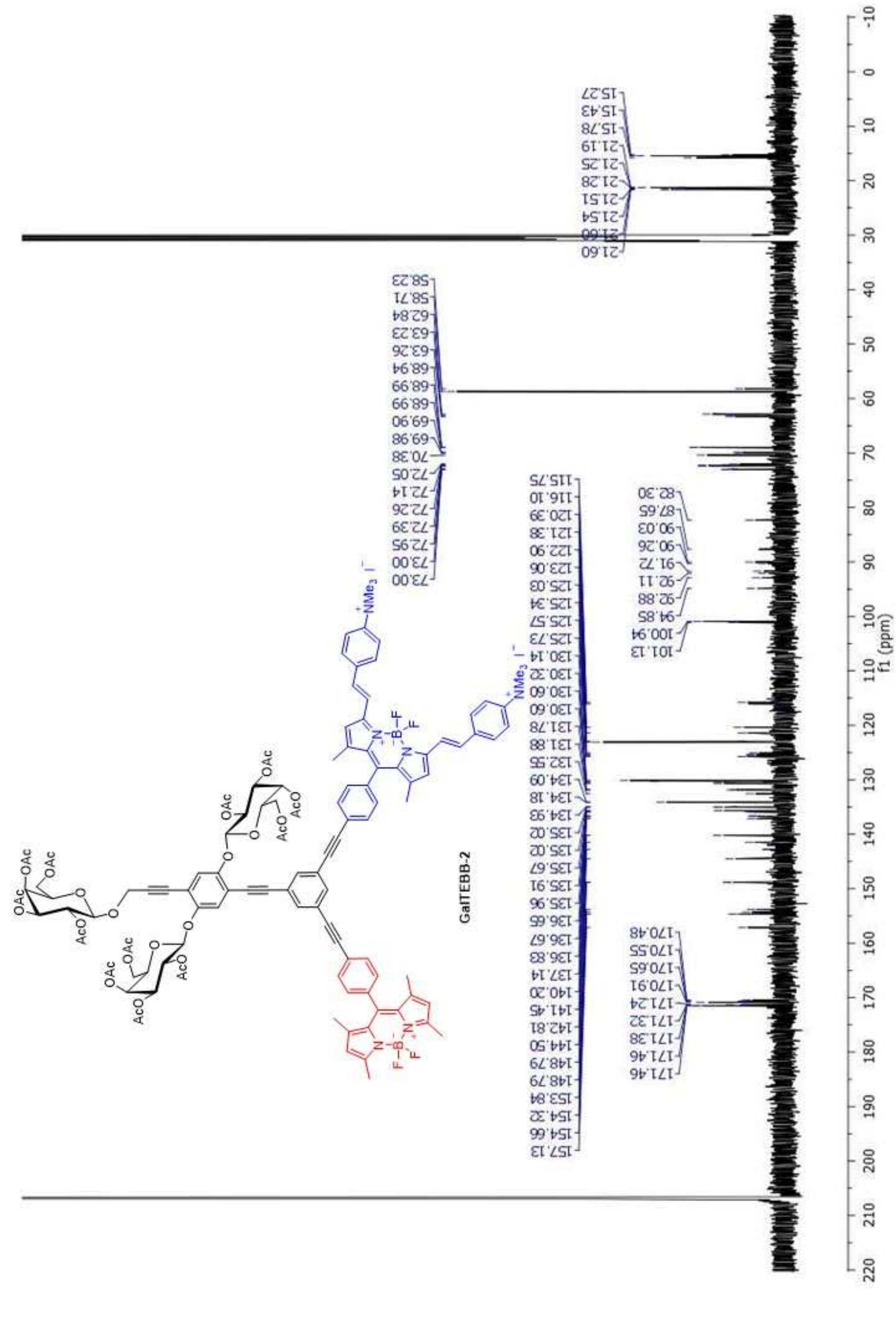

Figure S16.^{13}C-NMR compound **GalTEBB-2** in acetone-d_6

6. References

[1] Spicer, C. D.; Davis, B. G. *Chem. Commun*. 2013, **49**, 2747-2749.

[2] Bonaccorsi, P.; Aversa, M. C.; Barattucci, A.; Papalia, T.; Puntoriero, F.; Campagna, S. *Chem. Commun.*, 2012, **48**, 10550-10552.

[3] Nakamaru, N. *Bull. Chem. Soc. Jpn.*, 1982, **55**, 2697.

[4] Olmsted III, J. *J. Phys. Chem*. 1979, **83**, 2581.

[5] a) Serra, R.; Grande, R.; Butrico, L.; Rossi, A.; Settimio, U. F.; Caroleo, B.; Amato, B.; Gallelli, L.; de Franciscis, S. *Expert Rev Anti Infect Ther,* 2015, **13**, 605 613. b) Fischer A.J.; Singh S.B.; LaMarche M.M.; Maakestad L.J.; Kienenberger Z.E.; Peña T.A.; Stoltz D.A.; Limoli D.H. *Am J Respir Crit Care Med.*, 2021, **203**, 328-338.
